# Unlocking the musical brain: A proof-of-concept study on playing the piano in MRI scanner with naturalistic stimuli

**DOI:** 10.1016/j.heliyon.2023.e17877

**Published:** 2023-07-11

**Authors:** Alicja M. Olszewska, Dawid Droździel, Maciej Gaca, Agnieszka Kulesza, Wojciech Obrębski, Jakub Kowalewski, Agnieszka Widlarz, Artur Marchewka, Aleksandra M. Herman

**Affiliations:** a Laboratory of Brain Imaging, Nencki Institute of Experimental Biology of the Polish Academy of Sciences, 3 Pasteur Street, 02-093, Warsaw, Poland; b Department of Nuclear and Medical Electronics, Faculty of Electronics and Information Technology, Warsaw University of Technology, 1 Politechniki Square, 00-661 Warsaw, Poland; c 10 Murarska Street, 08-110 Siedlce, Poland; d Chair of Rhythmics and Piano Improvisation, Department of Choir Conducting and Singing, Music Education and Rhythmics, The Chopin University of Music, Okolnik 2 Street, 00–368 Warsaw, Poland

**Keywords:** Neuromusicology, Naturalistic paradigm, Error monitoring, MRI-Compatible keyboard, Music production

## Abstract

Music is a universal human phenomenon, and can be studied for itself or as a window into the understanding of the brain. Few neuroimaging studies investigate actual playing in the MRI scanner, likely because of the lack of available experimental hardware and analysis tools. Here, we offer an innovative paradigm that addresses this issue in neuromusicology using naturalistic, polyphonic musical stimuli, presents a commercially available MRI-compatible piano, and a flexible approach to quantify participant's performance. We show how making errors while playing can be investigated using an altered auditory feedback paradigm. In the spirit of open science, we make our experimental paradigms and analysis tools available to other researchers studying pianists in MRI. Altogether, we present a proof-of-concept study which shows the feasibility of playing the novel piano in MRI, and a step towards using more naturalistic stimuli.

## Graphical abstract

**Figure:**
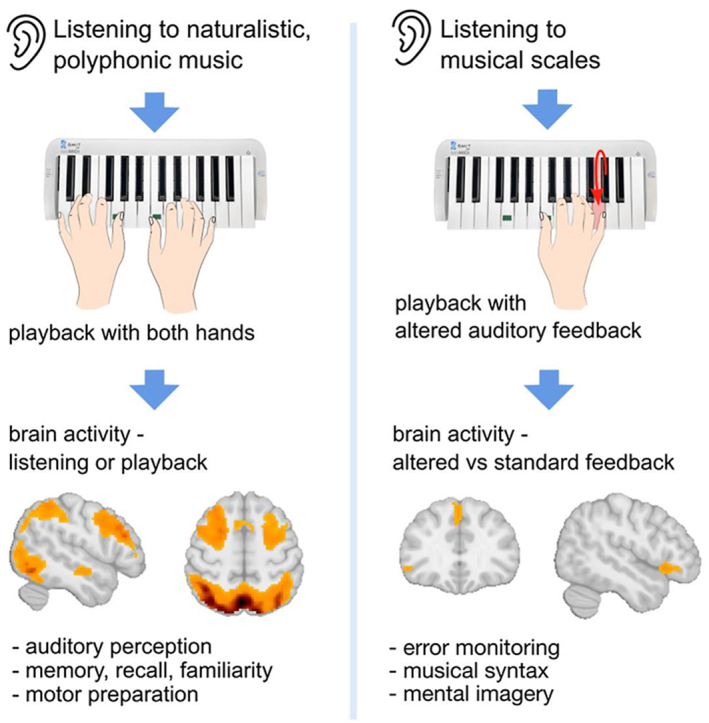

## 1 Introduction

The neuroscience of music is a broad study topic. It includes basic phenomena such as listening to and playing music, and music-based interventions in development [1], disease [2] and ageing [1]. Human musicianship can be used as a model for brain's predictive processing [3] or to study brain plasticity [4,5].

Yet, brain neuroimaging studies of human musicianship employing magnetic resonance imaging (MRI) often struggle with ecological validity, universality and replicability. These issues can result from the differences between various experimental environments or equipment and typical everyday conditions. For example, most musical instruments are not viable in the scanner environment because of their size and/or the use of ferromagnetic material. In studies on musical performance, the piano and similar keyboard instruments are frequently employed due to the instrument's versatility and popularity in musical education and practice. Western musical education includes piano training in almost all curricula, and due to its tempered and polyphonic nature, the piano is an attractive instrument to use for novices.

Despite the multitude of MRI studies on pianists or piano learning, only a few of them assess playing the instrument itself, suggesting that constructing an MRI-compatible piano-like keyboard is a nontrivial task. Several solutions were employed so far to investigate musicianship, including non-piano devices adapted for musical experiments, mock instruments, and some MRI-compatible instruments. In older studies, participants were asked to imitate playing the piano on a fibreglass/plastic board [6,7], or on electronic keyboards’ cases stripped of electronic components [8,9]. Of course, auditory feedback was not available in such solutions. Nevertheless, these studies gave important insights into the interplay between the auditory and the motor components of musical performance. Specifically, they have shown that the mere fact of listening to music can lead to activations in the premotor and supplementary motor areas, as well as playing without auditory feedback can activate the auditory cortices.

Two studies [10,11] used four-finger keypads designed for other purposes, which allowed for auditory feedback but did not resemble playing an actual piano. To study musicianship in a more naturalistic fashion, a couple of MRI-compatible keyboards with auditory feedback have been used [[12], [13], [14], [15], [16], [17]]. In these works, playing the piano invoked activation in the auditory-parietal-motor network, while improvisation added involvement in the prefrontal areas. However, little is known about the characteristics of these solutions, such as key velocity or the sound latency between the keypress and the sound. These aspects can influence how a musician performs and perceives the performance, which might influence the brain activation patterns measured with neuroimaging methods.

We are aware of three MIDI keyboard solutions already described in the literature solutions: the commercially available Hybridmojo LLC keyboard [18] and two prototype keyboards constructed at the Aarhus University [12] and the McGill Schulich School of Music [13]. So far, the descriptions of such instruments focused on the compatibility with MRI and basic functioning of the devices; additional information regarding instruments’ ecological validity is not provided. Hereunder we summarise the available details about these devices.

Hybridmojo LLC is an MRI compatible MIDI keyboard, commercially available on the market. This thirty-three key keyboard is free of ferromagnetic materials - springs are replaced with rubber and the case is made of plastic. Key press and key release are captured electrically.

The connection between keyboard and computer (or MIDI synthesiser) is obtained through the USB interface on a separate electronic device (with an implemented class of MIDI devices) connected to the keyboard via two cables with DB9 and DB25 connectors. The cables require compatible connectors on the patch panel of the MRI scanner's Faraday cage casing. The keyboard does not support the encoding of key press velocity. The keyboard was tested in a 3 T scanner and despite displaying safety in that environment it also tended to capture key presses caused by scanning sequence apart from the actual pressing [12]. These artefacts can be problematic when the behaviour of the participant is recorded and analyzed in an experiment. Thus, Jensen and his team created their own keyboard with optic transmission and optical key press detection based on a solution described in Hollinger et al. [13]. In this approach, the authors resigned from key press velocity detection, which requires additional adjustment and calibration. The key acts as a shutter that breaks the light transmission and its pressing is detected by a change in the light intensity. The approach is similar to the one presented in Hollinger et al. [13], with the difference that the light emitted from one fibre optic cable is reflected by the key surface and additional reflective surface and goes back to the fibre optic receiver cable. The solution used 25 keys, which results in 50 plastic fibre optic cables attached to the keyboard (25 receiver and 25 transmitter cables). These cables go through a waveguide in the Faraday cage and land in an electronic module equipped with a microcontroller, which transforms detected light intensity changes into corresponding MIDI standard notes on the 5 pin DIN connector output.

The biggest advantage of the solution provided by Jensen et al. [12] is the fact that it does not contain any metal elements. In addition, the key press detection is fully resistant to interference with MRI-scanner. The lack of interference between keyboard and MRI is also guaranteed by the keyboard construction. Another advantage is the use of the DIN connector for the MIDI signal output and the possibility of connecting it not only to the computer but also to the external MIDI synthesiser, which latency is smaller and much more stable than the latency of the MIDI subsystem in a desktop computer. Using the MIDI-thru functionality and connecting the keyboard simultaneously to the computer and the synthesiser allows for the registration of key presses also on the computer. Some limitations of this approach include that it lacks the ability to capture key press velocity and a large number of fibre optic cables reduces the usage comfort. In addition, registering key presses on a desktop computer requires using software that supports the MIDI standard (which is not supported by the Presentation software). This solution allows study participants to play an electronic keyboard while in an MRI scanner but does not provide the experimenters to manipulate keyboard output, such as a static and dynamic octave shift or dynamically programmed relation between key and the number of MIDI standard notes.

None of the three of the presented solutions provides information on the latency between pressing a key and the actual sound perception. Meanwhile, latency can be a substantial issue for performing musicians and should be kept as low as possible to ensure the instrument's ecological validity [19,20]. Apart from the three solutions described above, we are aware of two more MRI-compatible instruments, one used by de Manzano and Ullen [21] and the other by Kohler et al. [22]; however, even less information is provided about them. A comparison of the solutions and performance between different MRI-compatible keyboards is presented in Table 1.

**Table 1:** A comparison of the MRI-compatible keyboards with regards to used technology and performance.

Keyboard instrument	Magnetic field compatibility	Number of keys	Dynamic keys	Sensor type	Connection	Latency	Availability
Hybridmojo	Spurious activations @3 T	33	No	electric	USB	unknown	commercial
Hollinger et al. (2007)	3 T	24	Yes	fibre-optic	fibre-optic	unknown	prototype
Jensen et al. (2017)	3 T	25	No	fibre-optic	fibre-optic	unknown	prototype
De Manzano & Ullen (2012)	1.5 T	12	unknown	unknown	fibre-optic	unknown	prototype
Kohler et al. (2022)	3 T	27	unknown	unknown	unknown	unknown	unknown
fMRI MIDI (current study)	3 T	25	Yes	electric	wireless	99% < 20 m s	commercial

There are inherent limitations to functional MRI methodology in the case of auditory cognitive neuroscience resulting from the nature of the experimental environment: restricted space, supine position and scanner noise. Due to spatial restrictions of the MRI scanners, the register of the MRI-compatible keyboard has to be limited, and acoustic sound generation is not feasible due to scanner noise. Detailed considerations regarding managing scanner noise for acoustic experiments have been presented by Peelle [23]. With careful consideration of these restrictions, we constructed a set of fundamental requirements that an MRI-compatible keyboard instrument should satisfy to maximise its ecological validity and propose a device that satisfies these requirements.

Additionally, challenges related to experimental equipment and procedures pose limitations on the musical stimuli that can be used when studying musicianship. Typically, the stimuli are simple compared to what trained musicians are used to playing, and are frequently performed with the right hand only [14,16,17,24,25] in a fixed-finger position [14,25]. This approach limits the participant's movement in the scanner, reducing the chance of motion artefacts, simplifies the analysis of behavioural data acquired during playing, if any, but comes at the cost of further reduced ecological validity of an experiment. When designing experiments, researchers often have to choose between simpler, more controllable conditions, and more complex and ecological ones. Moreover, quantifying musical performance is not straightforward, and different approaches have been used for this purpose. Direct note-by-note comparison can be effective to measure correctness for simple melodies played with one hand [15,26] but falls short when advanced musical pieces are involved. In a more ecological setting, a performer might add or skip a note, which would render most of the following performance incorrect, if scored in the aforementioned way, even if otherwise it was played correctly.

Therefore, the aim of this paper was to address some of the limitations of previous music-production studies in the MRI environment. For this purpose, we present a complete experimental setup using a commercially available, wireless, MRI-compatible MIDI keyboard with 25 full-size, dynamic, programmable piano keys, a Presentation software plug-in to programme them, and a flexible algorithm for the analysis of a musician's performance of naturalistic stimuli. The keyboard has been developed in a collaboration between our institutions (Nencki Institute and the SMIT-lab company). Extensive details about its design and testing can be found in the supplement (S1–S3).

The two proof-of-concept experiments were designed to test, validate and showcase some of the features of our novel keyboard using experimental paradigms of playing naturalistic stimuli in the fMRI. In the first experiment, musicians played naturalistic musical pieces with both hands or only the right hand. In previous studies, musicians were limited to playing monophonically with only their right hand, even while improvising [12,[14], [15], [16], [17]]. We employed naturalistic musical stimuli that would be closer to the actual situation of playing the piano. Hence, our study includes pieces intended for two hands and in various musical keys. This step towards more ecological approaches makes the otherwise artificial situation of the study closer to the real executive practice. The listen-playback design was inspired by Chen et al. [15], where participants first listened to melodies and then attempted to replay them, during silent periods of the sparse-sampling paradigm. We expected this task to evoke responses related to music listening [9,[27], [28], [29]], motor control and performance monitoring [30], especially in the auditory-parietal-motor network (dorsal auditory stream). This includes the primary and secondary auditory cortices, the inferior parietal lobe, and the primary and secondary motor areas (for review, see Olszewska et al., 2021 ^4^).

In the second experiment, musicians played scales with the right hand with altered key-pitch mapping (altered auditory feedback) to showcase the unique feature of our device. We were inspired by other works on altered auditory feedback (AAF) [25,31] but wanted to preserve a similar design as in the first experiment (listen-playback) for participants' ease. For stimuli, we chose scales instead of musical pieces, as our pilot tests of the altered auditory feedback task indicated that AAF with musical pieces was too difficult to perform even for skilled musicians. Playing scales has been previously successfully used as a task in studies on musicianship [6,16], and is an automatised, simple task for professional musicians. Yet, contrary to previous studies, we did not want to shift the entire register of the keyboard (which is also possible in our setup), but instead, we wanted to test the keyboard's feature of altering key pitches on-the-fly. Perceptually, this would be more similar to making mistakes during natural piano playing, and we opted for this more naturalistic paradigm, because making errors during piano performance is an essential element driving the learning of new musical pieces or techniques. Music has an inherent temporal structure, which allows the human brain to expect following notes based on previous ones, utilising its fundamental capacity for prediction [3]. From previous studies using EEG and MEG we know that unexpected deviations from anticipated pitch, generating prediction errors, have a distinct neuronal correlate. This correlate can be measured as enhanced mismatch negativity, overlayed on the auditory evoked field elicited by the deviant sound, about 150–250 m s after its onset [32]. Such high temporal resolution is not applicable to fMRI; instead, we look for patterns of brain activity. These have been investigated in two previously mentioned works, one with the piano [25] and one with the cello or singing [31]. Based on this research, we expected that the processing of deviant sounds should activate the inferior frontal gyrus, the inferior parietal lobule, and possibly also the auditory temporal cortex and supplementary motor cortices. To measure whether the altered feedback was indeed similar to making an error, we checked for the post-error slowing effect in times between keypresses after AAF compared to before. Finally, we also introduce a novel way of scoring naturalistic musical performance. It is based on similarity to an errorless performance [[33], [34], [35]] using Levenshtein ratio [36] for melodic performance (the order of keys pressed), the rhythm-reproduction tapping-PROMS [37] approach for rhythmic performance, and can be easily scaled up for the analysis of naturalistic, polyphonic stimuli played on a tempered instrument, such as a piano of 60 keys or more.

## 2 Results

### 2.1 The fMRI-MIDI keyboard meets the design requirements

**Fig. 1:**
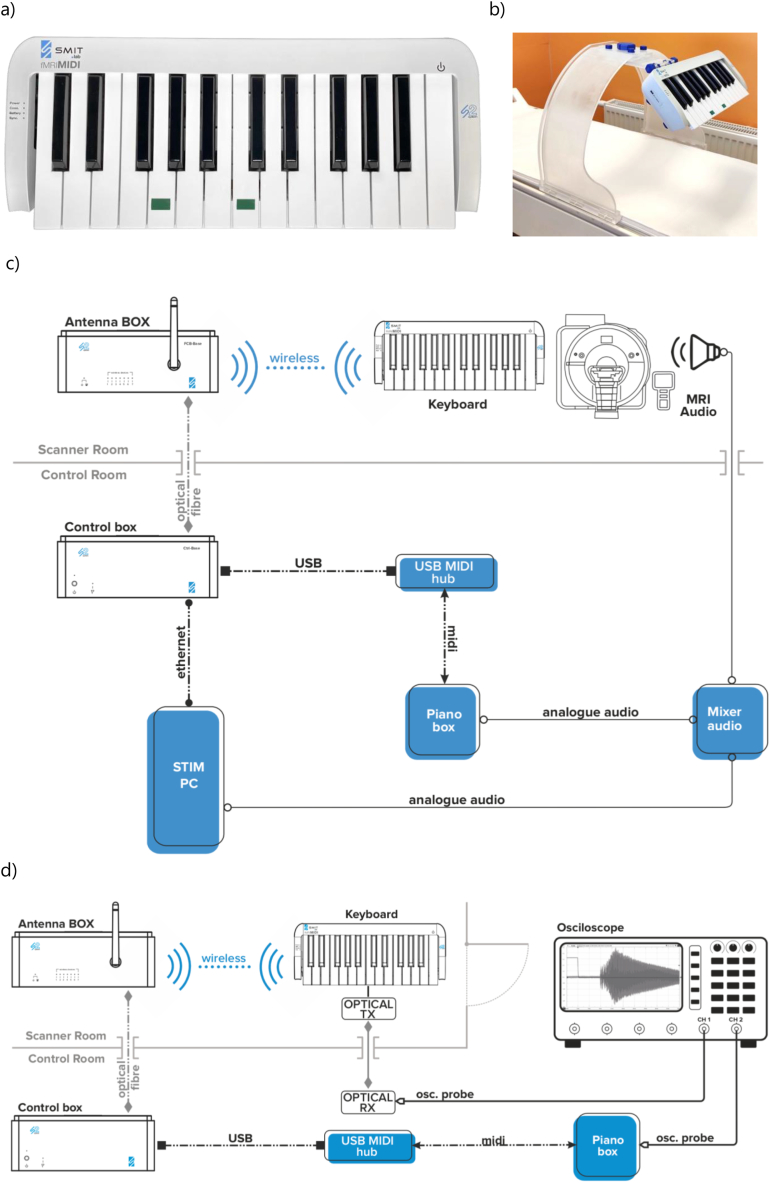
The wireless fMRI MIDI keyboard and the experimental setup. (a) A photograph of the keyboard; (b) the keyboard mounted on the keyboard stand for MRI experiment; (c) The information about key pressing (timing & force) is sent wirelessly to an *Antenna Box*, which in turn sends it to the control box via an optical fibre (♦). The *Control Box* communicates with the stimPC via ethernet (●), thus the timing and the force of key presses can be logged. The control box forwards the signal via USB (◾) to a USB MIDI hub, which translates it into MIDI encoding (▲), and sends it to the PianoBox which synthesises the sound in grand piano timbre. Analog audio (○) from the PianoBox (corresponding to key presses) and the stimPC (other auditory stimuli) is collected via an audio mixer and sent to the MRI audio system; (d) Measurement setup used to obtain latency distribution. An oscilloscope probe (Analog Discovery 2 by Digilent) was connected to a keyboard key on channel 1, via an intermediary fibre optic link (HFBR-0400 Series Avago Technologies), which was also the trigger source. Synthesiser output was connected to channel 2.

**Fig. 2:**
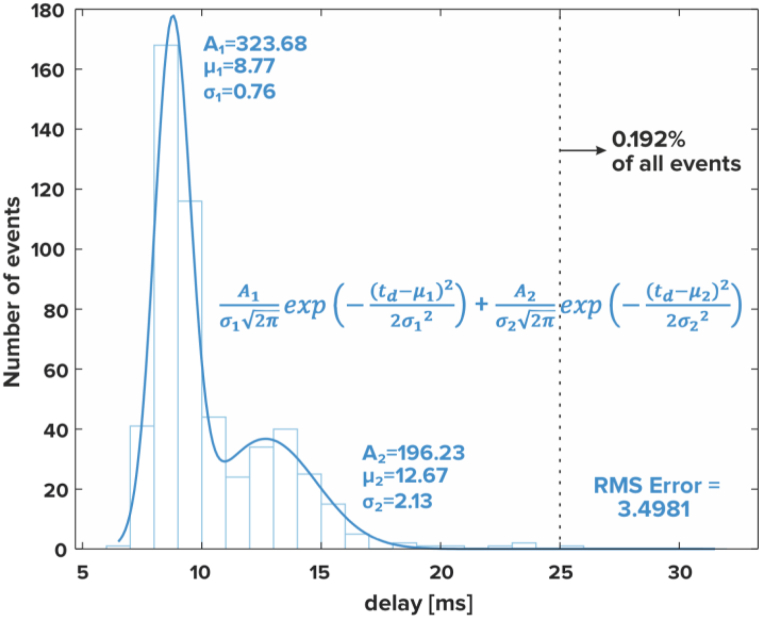
Latency distribution for the setup where the door to Faraday cage is closed and the MRI scanner is working with an EPI sequence.

Before we commenced research with human participants, we ensured that our device (Fig. 1a and b) meets design specifications, especially in the aspect of perceptual latency between pressing a key and the resulting sound in the experimental setting (Fig. 1c). Therefore, we conducted trial noise scans to check for any interference from the device on MRI scanner signal, and resistance scans to characterise keyboard performance in the MRI scanner. During the noise scans, our device emitted signal below the noise levels of the scanner's receivers (Supplement S2). During the resistance scans, all events were correctly recorded and no false events occurred, and we noted no interference of the magnetic field in the operation of our device. In the latency measurements (Fig. 1d), the distribution of the latency values was almost the same in all the three measurement setups: with the door to the Faraday cage open, closed with scanner off and closed with scanner on (Fig. 2). The measured distribution fits into two Gaussian models where the first mean value is 8.77 m s (∼82% of all events) and the second is 12.67 m s (∼18% of all events). Almost all events (99.808%) meet the maximum latency requirement (≤25 m s).

### 2.2 Proof-of-concept experiment

#### 2.2.1 Listening and playing naturalistic musical stimuli recruits brain regions related to memory, planning, attention, and auditory-motor processing

**Fig. 3:**
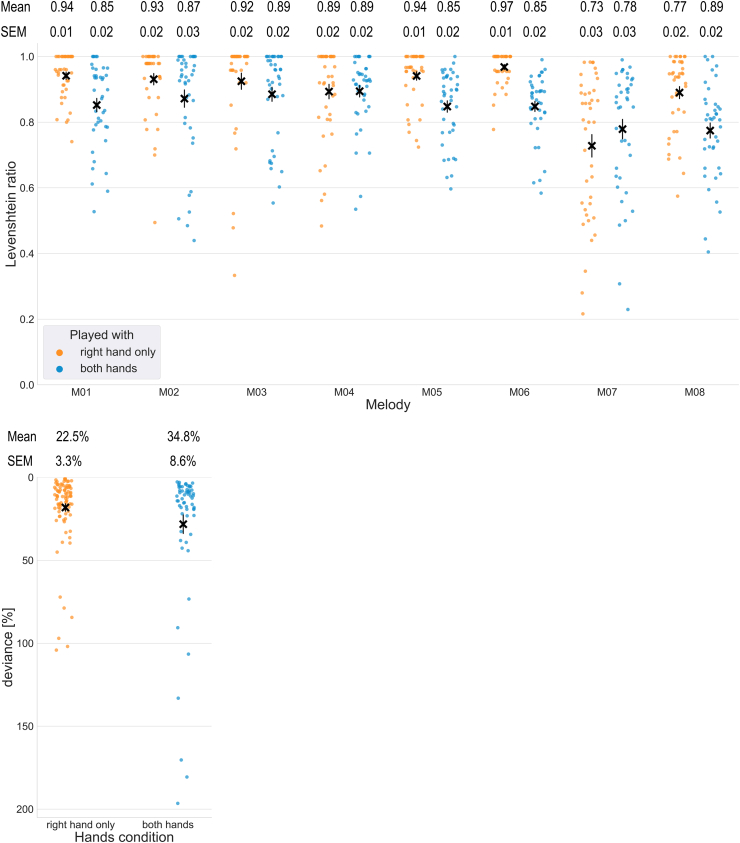
Participants' playback melodic (a) and rhythmic (b) performance across the melodies; ✖ indicates the mean performance score per melody, bars represent the Standard Errors of the Mean (SEM). Dots represent individual participants' performance scores; the mean and SEM of the scores per melody and hand condition are denoted above. For (a) the higher the score, the higher the melodic accuracy; for (b), score of 0 denotes expected timing.

All of the 20 study participants successfully completed this task and none was excluded due to excessive motion (see Methods for details). The mean melodic performance of the eight melodies used as experimental stimuli was high across participants (M = 0.873, SEM = 0.006, maximum score 1 = errorless performance), albeit not flawless (see Table 2 and Fig. 3a for details). The skewness (−1.56) and kurtosis (2.3) of the data fit within the boundaries of robustness (2.31 and 8, respectively) for repeated-measures ANOVA [38]. The test revealed a significant effect of both melody, hand condition, and their interaction in the Levenshtein ratio of actual versus errorless performance (Table 2). When playing with both hands, the participants performed significantly worse than when playing only with the right hand. Subsequent post-hoc analyses showed a significant interaction between the melody and hand condition for four melodies (M01, M05, M06 and M08). For rhythmic performance, 23.9% of trials were eligible for analysis (See Supplement S6, Fig. S6). The mean deviance from expected timing was 26.8% of the expected note duration (SEM = 3.9%). A *t*-test analysis revealed no significant difference between the right hand only and both hands conditions (p = 0.10, 95% CI = [−0.22, 0.02], T = −1.66, DF = 78.67, Chohen's d = 0.31, Fig. 3b).

**Table 2:** The results of repeated-measures ANOVA for the Levenshtein ratios from *Task I* and the Levenshtein ratios and mean time between keypresses from *Task II*.

Task I - Levenshtein ratio	DF1	DF2	F	p	effect size (η_p_^2^)
melody	7	133	14.71	<0.01	0.44
hands condition	1	19	24.57	<0.01	0.56
melody * hands condition	7	133	5.89	<0.01	0.24

**Task II - Levenshtein ratio**					

altered vs correct auditory feedback	1	5	1.68	0.21	0.10

**Task II - mean time between keypresses**					

before vs after AAF	1	15	5.25	0.04	0.26

Regarding fMRI results, the *listen* > *playback* contrast revealed a wide brain activation related to listening to stimuli but not playing (Fig. 4a, Fig. S8, Table 3). These included precuneus bilaterally and the supplementary motor cortices, extending bilaterally and into the regions of the superior parietal lobules, the angular and supramarginal gyri, cunei and occipital poles, and further into the bilateral cerebellum, fusiform gyri, hippocampi, superior/middle temporal gyri, and thalami, putamen and caudate nuclei, middle frontal gyri, and the medial frontal cortices. In the reverse contrast (*playback* > *listen*), we identified increased activation in the regions of the left postcentral/precentral gyrus, bilaterally in the central/frontal opercula, and in the right cerebellum (Fig. 4b, Table 3). Playing with both hands, when compared to playing with the right hand only, activated additional regions of the right sensorimotor cortex and contralateral cerebellum (Fig. 4c, Table 3).

**Fig. 4a:**
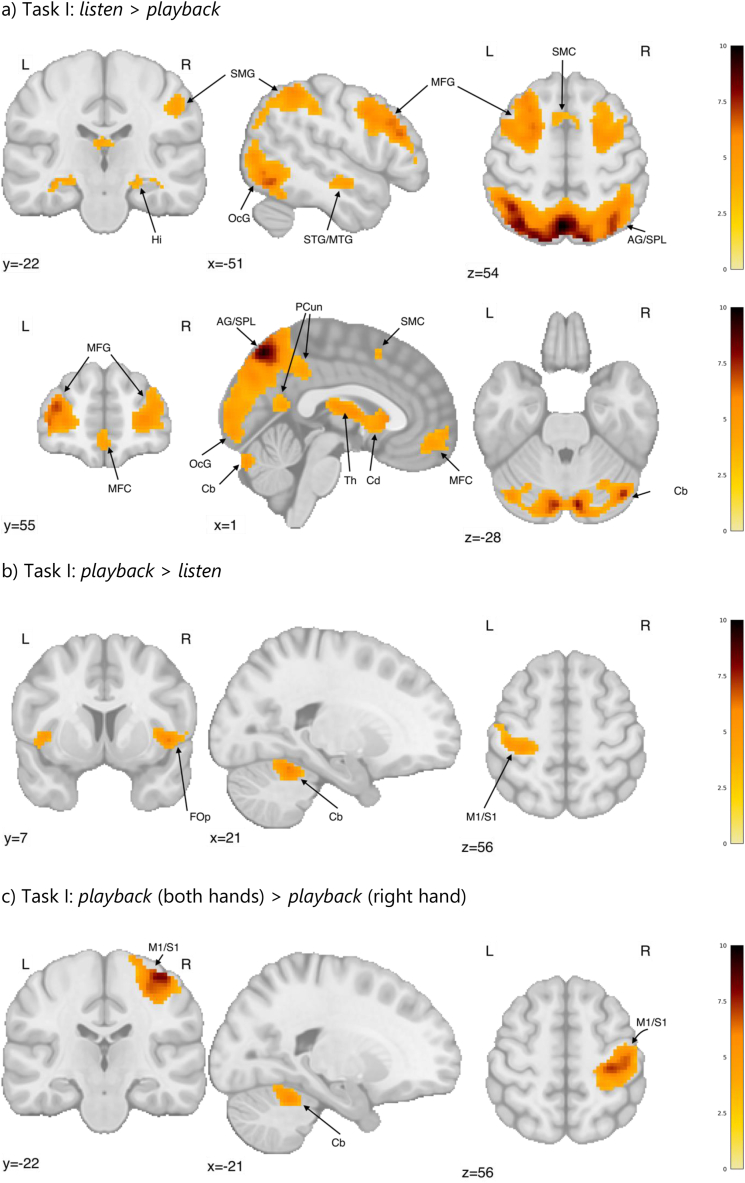
Statistical maps representing the contrasts for tasks I (a, b, c) and task II (d): (a) *listen* > *playback*, (b) *playback* > *listen*, (c) *playback* (both hands) > *playback* (right hand), (d) Increased brain activation in the altered auditory feedback condition compared to the standard auditory feedback condition. Colourbars represent test-statistics range. x, y, z - MNI coordinates AG - Angular Gyrus; Cb – cerebellum; Cd - Caudate Nucleus; FOp – Frontal Operculum; Hi – Hippocampus; M1/S1 - primary somatomotor cortex; MFC - Medial Frontal Cortex; MFG - Middle Frontal Gyrus; OcG – Occipital gyri; PCun - precuneus; SMC - Supplementary Motor Cortex; SMG - Supramarginal Gyrus; SPL - Superior Parietal Lobe; STG/MTG - Superior/Middle Temporal Gyrus; Th – Thalamus.

**Table 3:** Local maxima for the contrasts constructed based on neuroimaging data from *Task I*. Labels based on the Neuromorphometrics atlas from SPM12. A voxel-wise height threshold of p < 0.001 (uncorrected) combined with a cluster-level extent threshold of p < 0.05 (FWE corrected) was applied.

Region Label	T-values	X	Y	Z	extent
Listen > playback					
RL precuneus	10.16	−2	−68	54	17,179
L middle occipital gyrus	8.97	−32	−80	39	*
L superior parietal lobule	8.80	11	−78	52	*
L superior frontal gyrus	7.72	−24	10	66	3580
L middle frontal gyrus	7.29	−34	58	12	*
L middle frontal gyrus	7.19	−42	28	19	*
R middle frontal gyrus	7.31	41	30	22	2122
R superior frontal gyrus	6.61	26	5	62	*
R middle frontal gyrus	6.23	31	60	6	*
RL supplementary motor cortex	5.07	−6	15	49	153
R middle temporal gyrus	4.80	58	0	−19	148
R anterior orbital gyrus	4.78	31	38	−6	230
L superior temporal gyrus	4.78	−54	−8	−14	152

**Playback > listen**					

R cerebellum	8.50	8	−58	−14	544
L postcentral gyrus	6.56	−32	−28	72	554
R central operculum	6.20	46	8	−1	224
L frontal operculum	4.96	−42	8	2	110

**Playback (both hands)** > **playback (right hand)**					

R postcentral gyrus	8.41	38	−25	62	1026
R postcentral gyrus	7.88	46	−18	62	*
R precentral gyrus	5.44	26	−20	74	*
L cerebellum exterior	6.52	−14	−55	−16	309

* because of a large extent of the clusters (>1000 voxels), we decided to list secondary peaks.

**Fig. 4b:**
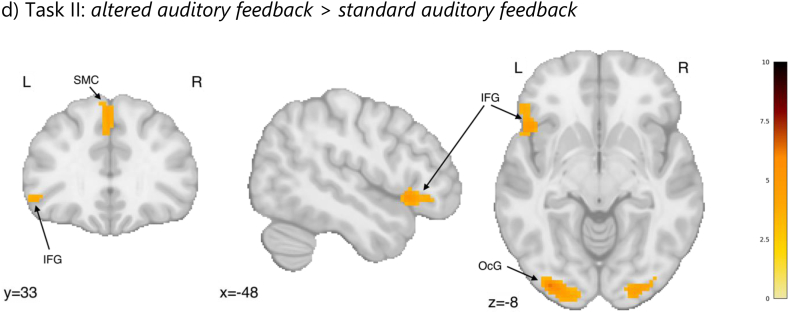
Statistical maps representing the contrasts for tasks I (a, b, c) and task II (d): (a) *listen* > *playback*, (b) *playback* > *listen*, (c) *playback* (both hands) > *playback* (right hand), (d) Increased brain activation in the altered auditory feedback condition compared to the standard auditory feedback condition. Colourbars represent test-statistics range. x, y, z - MNI coordinates AG - Angular Gyrus; Cb – cerebellum; Cd - Caudate Nucleus; FOp – Frontal Operculum; Hi – Hippocampus; M1/S1 - primary somatomotor cortex; MFC - Medial Frontal Cortex; MFG - Middle Frontal Gyrus; OcG – Occipital gyri; PCun - precuneus; SMC - Supplementary Motor Cortex; SMG - Supramarginal Gyrus; SPL - Superior Parietal Lobe; STG/MTG - Superior/Middle Temporal Gyrus; Th – Thalamus.

#### 2.2.2 Altered auditory feedback activates error-related processing in the left inferior frontal gyrus

**Fig. 5:**
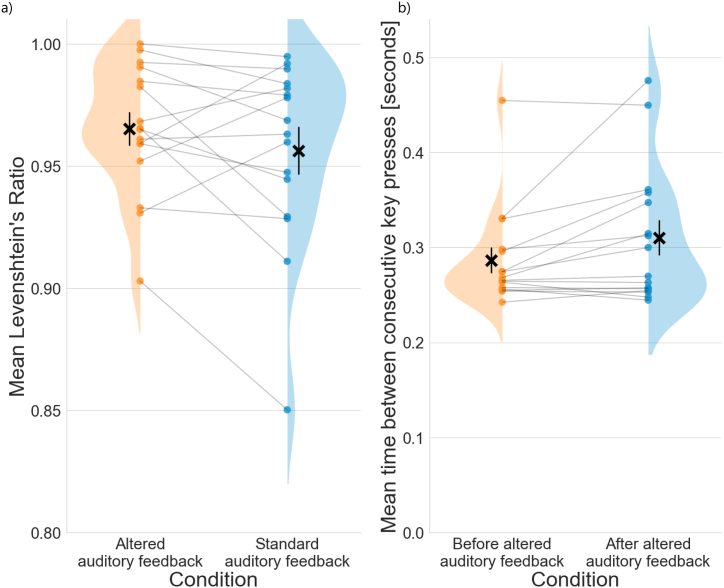
(a) No significant differences in the performance of scales between the altered auditory feedback (orange) and standard feedback (blue) conditions as measured with the Levenshtein ratio; please note that the vertical scale is expanded and starts from 0.8. (b) A significant slowing in the mean time between consecutive key presses for keys preceding (orange) and succeeding (blue) the altered auditory feedback target key. ✖ represent the means; error bars represent the Standard Errors of the Mean (SEM); dots represent individual means per participant.

Of the 20 participants, 17 completed this task. One participant was excluded due to excessive head movements, resulting in the final sample of 16 participants for this task. The mean performance of the scales was very high (Levenshtein ratio ⩾ 0.85) across study participants. No significant differences were found in the performance of playing scales between the altered auditory feedback and standard feedback conditions (Table 2, Fig. 5a). However, we observed a significant difference in the mean time between consecutive key presses for keys before and after the altered auditory feedback, indicative of port-error slowing (Table 2, Fig. 5b).

Playing with altered auditory feedback evoked increased activations in the visual cortex bilaterally, the left inferior frontal gyrus and left supplementary motor cortex, compared to playing with standard auditory feedback (Fig. 5, Table 4).

**Table 4:** Local maxima of the significant clusters and their anatomic labels in the altered > standard auditory feedback contrast. Labels based on the Neuromorphometrics atlas from SPM12. A voxel-wise height threshold of p < 0.001 (uncorrected) combined with a cluster-level extent threshold of p < 0.05 (FWE corrected) was applied.

Region Label	T-value	X	Y	Z	cluster size
L inferior occipital gyrus	6.27	−34	−90	−8	303
L inferior frontal gyrus	5.20	−49	20	−6	125
R occipital fusiform gyrus	5.16	26	−90	−14	171
L supplementary motor cortex	4.49	−2	30	52	300

## 3 Discussion

We aimed to present and validate a complete framework for studying pianists in MRI using naturalistic stimuli in a highly replicable manner. To achieve that, we created a list of fundamental minimal requirements that have to be satisfied by a keyboard instrument (See Methods: Design of the novel MRI-compatible keyboard) and built a device that satisfies these requirements. Moreover, we designed a proof-of-concept experiment which uses the device in two different tasks. In the first task, we used naturalistic polyphonic stimuli, some with polyphonic features, to show the feasibility of the experimental setup. In the second task, we further showcased the use of our instrument's functionality to change key-sound bindings on-the-fly in our altered auditory feedback paradigm to study how musicians process performance-related errors. These procedures and the scripts used to analyse data as well as the preprocessed dataset are freely available via an open-science platform.

Our fMRI MIDI keyboard provides considerable improvements over already available solutions, such as the prototype designs proposed by others [12,13] or the commercially available instrument [18]. In the preliminary tests, the device not only met minimal technical requirements but also did not generate spurious activations. Apart from satisfying fundamental requirements, the device introduces a couple of novel features compared to previously described designs. First, the keys are dynamic, which means that the velocity of keypress is translated to sound intensity, similarly to acoustic instruments. The velocity gain can be regulated to adapt to various experimental settings. Second, the pitch and velocity of each key can be adjusted on-the-fly during the experiment using a dedicated plugin, which allows for various study designs. For our proof-of concept experiment, we chose one such design, the altered auditory feedback task where single notes are changed while the participants are playing. Third, the wireless design improves functionality and the user experience, and the keyboard stand provides means for positioning the keyboard in a manner that can increase pianist's comfort during an experiment. Playing the piano is primarily a motor task and as such the change of posture required to play in an MRI scanner (from sitting to supine) can influence the performance of a musician. Finally, the data we provide on the reliability and latency of our device, and the experimental procedures in an open science format, can be used by other researchers to coordinate the environment of their studies to improve reproducibility of research on musicianship. With this information, close replication of our experimental conditions should be possible.

Furthermore, we illustrated how our novel MRI-compatible instrument can be used in a proof-of-concept experiment using two different tasks. The first task employed naturalistic polyphonic music stimuli. To our knowledge, this is the first study to include complex, polyphonic stimuli played by both hands in the MRI environment.

The more complex musical stimuli provide additional challenges for the analysis of behavioural data. Building on previous solutions, we decided to use a comparison between the actual and an errorless performance with Levenshtein ratio as a measure of musical performance. We conveniently used 25 capital letters of the Latin alphabet (A-Y) to encode the 25 keys of the C3–C5 register. This encoding can be easily extended to include more keys, e.g. using 26 capital letters (A-Z), adding 26 small letters (a-z), digits (0–9) and 12 punctuation marks (.,?!+- = /*@#$) can represent all 74 piano keys over 6 octaves. As long as each key is uniquely represented by a single character, the Levenshtein distance between the errorless and the actual performance can be calculated, making this approach a simple and flexible solution for such stimuli. The introduction of space character as a separator for simultaneously occurring sounds allows for the encoding of complex stimuli, including polyphonic features. This score is a useful behavioural measure which can be used to quantify performance differences between study participants or measure task difficulty or its execution. Using this method in *Task I*, we have shown that the performance was not uniform across the musical pieces, and that playing with both hands was more difficult to the participants than playing with only the right hand. For the second task, we have demonstrated no significant differences (no excess errors in performance) due to altered auditory feedback compared with standard feedback when playing scales with the right hand. These results illustrate that it is feasible to measure performance of naturalistic musical stimuli and that stimuli design and complexity can influence task performance. Moreover, it is possible to measure individual differences between participants, which can be used e.g. to exclude participants or trials with poor performance.

Levenshtein's ratio assesses only information related to the succession of keypresses, regardless of their rhythmic accuracy. To compare the rhythmic accuracy of participants' performances, we adopted the recently developed rhythm reproduction tapping-PROMS method [37], which compares actual rhythm reproductions to an errorless template, paralleling the approach to use Levenshtein's ratio for melodic performance. Using this method, we have shown no significant difference in rhythmic accuracy between playing only with the right hand and with both hands in Task I. However, the method can only be applied for trials where the number of keystrokes exactly matches the template. In the original experiment, which assessed this method for tapping of rhythms of various complexity [37], only 10% of all trials had to be removed due to this restriction, whereas in our case 76% of trials had to be removed. Thus, we show that the rhythm reproduction analysis can be applied to complex musical stimuli, with the limitation that only certain trials can pass the analysis eligibility criterion. Because accidental keypresses and mistakes are often the cause of increased number of notes played by the participants, a further refinement of this method would allow for a more complete picture of the participants' rhythmic performance.

The neural activity related to the playback task for the naturalistic stimuli (*Task I*) revealed a widespread brain activation in the *listen* > *playback* contrast. Similarly to previous MRI studies on music perception, we identified an activation in the superior and middle temporal gyri, typically associated with auditory processing related to music [9,[27], [28], [29]]. However, our task was not just auditory perception, but part of a *listen-playback* dyad. The *listen* trials were followed by the *playback* trials in which participants were asked to play back the listened fragments of melodies. In line with this task, we found an activation in areas involved in memory, planning and visuospatial attention, likely reflecting the effort participants made to recognise and memorise the fragment of the melody to play it back during the following *playback* trial. The medial frontal cortex as well as the left angular, middle and superior temporal gyri are associated with musical familiarity [39]. An increased activation in the right hippocampus, left precuneus and bilateral middle temporal gyri may be linked to successful melodic memory retrieval [40]. Similarly, previous studies linked the thalami, putamens, superior temporal and precentral gyri, as well as the left supramarginal gyrus activations to recognition of familiar melodies across different lifespans [41]. The supramarginal gyri bilaterally have also been shown to take part in pitch and rhythm memory [42]. A meta-analysis indicated the thalamus and the supplementary motor area as some of the brain regions related to familiarity in music [43]. The involvement of these regions in the *listen* condition can reflect its aspect of memory retrieval and recognition of familiar melodies. We also identified increased activation in regions of the motor network, including the supplementary motor cortex, the caudate nuclei and the cerebellum which might be related to movement planning, corresponding to the preparation aspect for the following *playback* condition [44]. The caudate nuclei are typically associated with motor function and related to rhythmic aspects, also in listening [45]. The involvement of the middle frontal gyrus might also reflect the preparation of movement related to auditory cues [46]. Alternatively, the activity of the motor network might indicate the familiarity of the participants with the material. In agreement, in previous studies, listening to a learned melody invoked activation in the premotor and supplementary motor cortices despite a lack of movement in both novice [29,47] and skilled performers [6,9,48]. Altogether, the *listen* > *playback* contrast revealed widespread brain activation related to memory retrieval and recognition of familiar melodies, and preparation for a motor task in the near future.

The reverse contrast (*playback* > *listen*) showed increased activation in regions of the motor network, including pre- and postcentral gyri as well as cerebellum. The left precentral and postcentral gyri are typically associated with the sensorimotor control of the right hand [30]. The right cerebellum and the bilateral opercula are involved in motor control and performance monitoring [49,50].

Finally, we identified increased activation related to the motor control and execution in the left hand (left cerebellum, right motor cortex) when subtracting playing with the right hand from playing with both hands. Although the stimuli differed acoustically depending on how they were played, we did not find any differences in activation in the auditory areas related to this difference.

In the second task, we contrasted playing musical scales with the right hand using standard and altered auditory feedback (an error imitation). While no significant differences were found in playback performance, a small but significant post-error slowing effect was found for the keys succeeding the altered auditory feedback compared to the keys preceding it. This is similar to the post-error slowing effect found in the stop-signal tasks - a common measure of prepotent response inhibition [51,52]. Therefore, despite no differences in musical performance between standard and altered auditory feedback, this paradigm captured a typical behavioural signature of error-making: a predictable deceleration in response time thought to index performance monitoring [53].

In line with the behavioural findings, we identified a neural signature of error-related activity in the frontal and occipital areas. This pattern of activations suggests that musicians perceive and react to the auditory feedback incongruent with their expectations differently than when playing with standard auditory feedback. Importantly, our results show a greater involvement of regions typically engaged in fine motor control and error monitoring, such as the inferior frontal gyrus and the supplementary motor cortex [[54], [55], [56]], during the condition with altered auditory feedback. The activity of the inferior frontal gyrus and the supplementary motor cortex have also been linked to altered auditory feedback in previous studies using slightly different paradigms [25,31]. In these studies, the whole auditory feedback performance was either shifted or randomised, whereas in the current investigation we altered only a sound of a single key to a sound of a neighbouring key, providing a less pronounced mismatch. Despite the differences in paradigms, we were able to replicate the involvement of the regions responsible for the perception-action coupling in the auditory system related to musical processing [30] in AAF. Furthermore, the inferior frontal premotor regions identified by AAF have been associated with the perception of deviant musical patterns [57], musical syntax [58,59], and compensation of pitch perturbations [31]. The increased activations observed in the occipital lobe might relate to the imaginative element of musical performance, as has been previously identified in studies on imagined performance [8]. Possibly, when not able to use visual feedback, musicians try to imagine their performance in order to understand the source of the perceived incongruence and monitor their behaviour. Altogether, these outcomes point to the involvement of networks associated with mental imagery, motor control and error monitoring during playing with altered auditory feedback in professional musicians.

In the future, it would be interesting to incorporate the task performance scores into fMRI analysis, in order to relate the observed patterns of activity to the behaviour of participants. Such tasks should be designed with a greater variability in the difficulty of the stimuli or participants’ skills. Future directions could also include an AAF paradigm with more complex, naturalistic stimuli.

## 4 Limitations of the study

There are certain limitations to our experimental design. First, we acknowledge that the latency of our keyboard can be noticeable to some well-trained, latency-sensitive musicians. The ability to perceive latency between the action and the perception varies between individuals, and some musicians might find the 20 m s perceptible. Although we did not gather feedback on this aspect in a formal manner, only one of the participants mentioned that the latency was noticeable. Second, in our experiment, the musicians could not see their hands while in the MRI scanner. It is possible that the behavioural performance of the musical pieces could be higher, and the difference in performance between the *both hands* and the *right hand* playback conditions could be smaller, if the musicians could use visual feedback to guide their performance. It is also likely that the activity of the somatosensory areas would be diminished in such a case. This drawback can be alleviated with a different mirror design; our mirror allowed the musicians to view cues on the screen behind their heads instead. Moreover, the supine position in the fMRI scanner is not optimal for playing the piano, which is typically played sitting down. This limitation, however, is inherent to the MRI environment.

One of the difficulties regarding auditory cognitive neuroscience with the use of fMRI methodology is MRI scanners noise, which interferes with the stimuli. To mitigate this issue, we opted for an interleaved silent steady-state paradigm (ISSS [60]), which limits the scanner noise substantially, although not completely. The ISSS paradigm itself poses certain limitations, as it requires precise timing of the stimuli, imposes a limit on the duration of the stimuli, and requires a slight modification to the standard analysis pipeline. Nevertheless, we were able to successfully implement the ISSS paradigm in our experiments. Future experiments aiming to use longer auditory stimuli might require different approaches.

## 5 Conclusion

Playing music requires the involvement of multiple brain networks, such as memory, sensorimotor, and the action-perception coupling between the sensory (auditory, tactile, proprioceptive) and motor domains [30]. For the first time, we show an interplay between music listening and playing using naturalistic, polyphonic stimuli. The proposed experimental setup and tasks using a novel, highly ecological MRI-compatible keyboard, revealed the involvement of multiple brain networks in the *listen-playback* task, including regions related to auditory processing, music familiarity and memory, multisensory integration, movement planning, execution and performance monitoring. Additionally, music as a phenomenon of soundscapes changing in time is an excellent tool to study the predictive coding apparatus of the human brain [3]. When a musician performs, they anticipate future sounds and movements based on learned sequences and associations. In the *altered auditory feedback* task, we identified regions related to motor control and error processing, as well as multisensory integration and imagery. This way, we were able to investigate what happens when expectations are violated and the prediction error increases. These results support high ecological validity of the instrument.

We present a ready, ‘off-the-shelf’ based approach, feasible for studying pianists in MRI. The device we describe is commercially available together with an open-source set of parameters such that the experimental conditions can be easily matched. Additionally, we provide software tools for our experimental procedures and the analysis of both the sparse fMRI data and the behavioural piano performance data on an open science platform. We hope that researchers conducting future studies can build on our solutions towards more ecologically valid and reproducible research on musicianship.

## 6 STAR methods

### 6.1 Key resources table

**Table:** 

REAGENT or RESOURCE	SOURCE	IDENTIFIER
**Deposited data**		
Data supporting main findings	This paper	https://osf.io/4rkxu/
**Software and algorithms**		
Presentation (Version 20.1, Build 12.04.17)	Neurobehavioral Systems, Inc., Berkeley, CA, www.neurobs.com	RRID:SCR_002521
MATLAB Version R2019b (data analysis)	https://www.mathworks.com/products/matlab.html	RRID:SCR_001622
SPM12	https://www.fil.ion.ucl.ac.uk/spm/	RRID:SCR_007037
fMRIPrep Version 21.0.0	https://fmriprep.org	RRID:SCR_016216
Python Programming Language Version 3.10	Python software foundation, http://www.python.org/	RRID:SCR_008394
pingouin Version 0.5.1	https://pingouin-stats.org/	RRID:SCR_022261
Code	This paper	https://osf.io/4rkxu/
**Other**		
fMRI MIDI keyboard	This paper, https://smit-lab.eu/	N/A

### 6.2 Resource availability

#### 6.2.1 Lead contact

- Further information and requests for resources and reagents should be directed to and will be fulfilled by the Lead Contact, Alicja Olszewska (a.olszewska@nencki.edu.pl).

#### 6.2.2 Materials availability

- The MRI-compatible keyboard is a commercial product which can be purchased from https://smit-lab.eu/
- Due to copyright restrictions, the stimuli are available on request for research or educational purposes.

#### 6.2.3 Data and code availability

- Preprocessed behavioural and neuroimaging data have been deposited at the Open Science Framework and are publicly available as of the date of publication. DOIs are listed in the key resources table.
- All original code has been deposited at the Open Science Framework and is publicly available as of the date of publication. DOIs are listed in the key resources table.
- Any additional information required to reanalyze the data reported in this paper is available from the lead contact upon request.

#### 6.2.4 Experimental model and subject details

A total of 20 healthy, right-handed female students (age range 19–26 years, M = 21.9, SD = 2.1) were recruited, which is a typical group sample size in recent publications on pianists [61,62]. All participants completed formal secondary music education on a keyboard instrument (piano, harpsichord, pipe organ, accordion) and had a total of 11–20 years of experience with keyboard instruments (M = 15.2, SD = 1.88). All were native Polish speakers and had normal or corrected-to-normal vision, normal BMI, lack of psychiatric and neurological illness, and unimpaired hearing. Two of the participants reported having absolute pitch. All participants provided written informed consent and were compensated for their time. This study was approved by the Research Ethics Committee at the Institute of Psychology of the Jagiellonian University, Kraków, Poland and the experiment has been carried out in accordance with The Code of Ethics of the World Medical Association (Declaration of Helsinki).

### 6.3 Method details

#### 6.3.1 Design of the novel MRI-compatible keyboard

To play an instrument during a fMRI scan, the device has to be safe, sized appropriately so it fits in the narrow environment of the scanner borehole, and, preferably, perform as close to a typical musical instrument as possible. Therefore, we created a set of fundamental requirements for the keyboard.

- 3 T compatibility - the device is safe and performs appropriately in the 3 T magnetic field, widely used in MRI research environments worldwide;
- outer dimensions of a maximum of 600 mm (fits into most scanner boreholes);
- at least 24 keys (two full octaves);
- full-size keys (160–165 mm per octave) - there is no standardised measure for piano keys, and their dimensions can differ between manufacturers;
- dynamic keys (keypress velocity recording);
- maximum sound latency (key- > headphones) 25 m s ^19^;
- MIDI interface and an interface for communicating with commonly used research software.

With this in mind, we designed a MIDI keyboard that, apart from meeting the fundamental requirements, also maximises user comfort and usage flexibility. For these reasons we chose a wireless design, and we imposed that the device has to operate within a minimum of 4 m from the receiver station for at least 12 h supplied from a battery, with a maximum charging time of 12 h. Additionally, we designed a dedicated keyboard stand, which allows for position angle and distance adjustments to maximise participants comfort in the scanner.

The keyboard extends the already commercially available S2 system platform (SMIT-Lab, http://smit-lab.eu/) used in our lab. This platform provides hardware and software environments for the equipment for fMRI experiments, including wireless response pads and synchronisation. It communicates with a (Windows) PC via ethernet and a dedicated desktop application. For compatibility reasons, translators from the Ethernet network to devices such as serial ports or HID devices are built into the S2 system and available via USB. This system can be used with any modern software, such as E-Prime (Psychology Software Tools, Inc. www.pstnet.com), PsychoPy (Open Science Tools Ltd, www.psychopy.org), etc. A dedicated plug-in for the control of the fMRI MIDI keyboard is available for Presentation software (Neurobehavioral Systems, Inc., Berkeley, CA, www.neurobs.com). From the electronic and software standpoint, the MIDI keyboard is simply a response pad with a larger number of keys and more extensive functionality.

The keyboard dimensions are 395 × 62 × 160 mm (width × height × length). Full-sized keys (163 mm octave width) were used to bring the fMRI keyboard user experience close to playing a real instrument. To adapt the keyboard size to the majority of MRI scanners it was limited to 25 keys (2 octaves + 1) (Fig. 1a). The keys were designed as levers with rubber springs, analogically to the majority of MIDI keyboards available on the market. Every key was equipped with two electrical contacts. When the key is pressed, both contacts are closed with a delay relative to one another, which depends on the key presses velocity. The measured delay is then converted by software to the sound intensity. This allows for the adjustment of the keyboard's articulative characteristics to another keyboard, for example, a training keyboard. Implemented dynamic sound articulation is similar to solutions utilised in professional keyboards commonly used by musicians.

We recognise that the comfortable and repeatable position of the user is important, hence the keyboard was equipped with universal fixing holes, such that it can be fixed e.g. to the dedicated stand.

The keyboard is an element of the wireless network of the S2 system by Smit-Lab. The battery allows at least 12 h of operation. Registered events consist of key presses (including keypress velocity) and releases. Each event from a wireless device is time-stamped by a hardware clock with 1 m s accuracy. Events are sent wirelessly to a receiver placed in the MRI scanner room (Fig. 1c). Subsequently, they are transmitted via fibre optic bus outside of the Faraday cage to the *Control box* device, which makes the event available via USB bus in a USB MIDI standard. Additionally, each event is sent via Ethernet and can be logged or handled by the control computer (here: a PC with the Presentation software using a special plugin). Dedicated application or Presentation software is also able to dynamically configure the keyboard during the experiment, for example, modify the octave shift or assign a specific MIDI note to each key at any moment.

*Control Box* implements MIDI standard as another interface of a USB composite device, hence when using a hardware synthesiser, it might be necessary to use a USB MIDI hub. This hub would make the MIDI events available via DIN connector, as not all MIDI synthesisers support composite devices.

More information about the development and software can be found in Supplement part S1.

#### 6.3.2 Device testing

The safety of our device in an MRI environment is guaranteed by design - the lack of any ferromagnetic elements. Nevertheless, the fMRI MIDI device contains electronic parts, therefore it was tested in 1.5 T (Siemens Avanto) and 3 T (Siemens TrioTim) scanners prior to its use in our proof-of-concept experiment.

Interference was tested in a standard, nominal position and in the centre of an MRI scanner in 3 orthogonal positions and with the use of the Body coil and the RF Noise sequence (TR = 30 m s, TE = 0.35 m s, Averages = 30, Measurements = 30, Vector Size = 512, developed by the Martinos Centre for Biomedical Imaging at Massachusetts General Hospital). Noise scans were performed by simply opening the MRI receiver without employing RF or gradient pulses.

Our device resistance was tested during EPI, DTI, T1 and DWI sequences on the same scanners and under the following test conditions.

- device in a central position, keys not pressed, the occurrence of false events is being observed;
- device in a central position, keys are pressed cyclically (with a period of about 1 press every 2 s), detecting if all events occurred.

The test lasted about 3 min for each sequence and condition.

Wireless S2 devices use multiple media and buses: radio bus, optical bus, Ethernet, USB and MIDI. Because of that, a small delay between key press and generation of the MIDI signal is expected. For optimal performance when used by professional musicians, we assume the delay to be no more than 25 m s [19]. The influence of various factors on delay can be hard to calculate, so the best way is to measure it.

Therefore, after a prototype was constructed, it was tested in real-life scenarios (Fig. 1d). A single key was configured to generate MIDI note number 110, as high-frequency sound helps to identify the start of the signal. 16,384 samples (sampling frequency 306,748 Hz) of both channels were written to the file with each trigger of the oscilloscope. We registered more than 500 events of keypressing in three scenarios.

1. The door to the Faraday cage is open,
2. The door to the Faraday cage is closed and then the MRI scanner is not working,
3. The door to the Faraday cage is closed and the MRI scanner is working with EPI sequence.

To obtain latency distribution, we calculated latency values using a custom MATLAB 2019b (Mathworks, http://www.mathworks.com) program, with latency defined as the time between the trigger event on channel 1 and the generation of the MIDI sound from the synthesiser.

#### 6.3.3 Experimental setup

We set our keyboard to represent the C3–C5 register in the standard A = 440 Hz tuning. To help the participants orient themselves on the keyboard without looking, we marked the G3 and the C4 keys with convex stickers, which facilitate tactile discrimination (to minimise movement). The experimental procedures were delivered using Presentation® software (Version 20.1, Build 12.04.17, Neurobehavioral Systems, Inc., Berkeley, CA, www.neurobs.com). The signal from the ControlBox was sent via USB to a MuMIDI device from Microsonic Solutions Ltd, which translated it into a MIDI signal, which in turn was synthesised in grand piano timbre via a PIANOBOX synthesiser (MIDITECH INTERNATIONAL). The sound from the Presentation® software and the PIANOBOX was combined via mixer and delivered to the participant over piezoelectric MRI-compatible headphones (MR confon GmbH, Magdeburg, Germany, www.mr-confon.de).

During the latency measurement, an oscilloscope probe (Analog Discovery 2 by Digilent) was connected to a keyboard key on channel 1, via an intermediary fibre optic link (HFBR-0400 Series Avago Technologies), which was also the trigger source. Synthesiser output was connected to channel 2. A schematic diagram of both setups can be found in Fig. 1c&d.

#### 6.3.4 Proof-of-concept experiment

##### 6.3.4.1 Experimental procedures

All of the tasks were performed in a supine position in an MRI scanner. The MRI MIDI keyboard was placed on a dedicated support stand. Beads-filled cushions were used to support participants’ elbows in a comfortable position. The participants could not see their hands during the scan and were asked to refrain from trying to look at their hands or the keyboard, limit unnecessary movements, and concentrate on the visual cues. The music score was not presented to the participants during the fMRI tasks.

Before scanning, all participants underwent a roughly 20-min familiarisation procedure, when they could practise in the position required to play in the scanner and learn how to perform fMRI tasks. During the familiarisation procedure, the tasks were abbreviated and made use of separate equipment (mock scanner, MIDI keyboard of 25 keys, headphones), but the same stimuli.

Behavioural data acquired during scanning encompassed the timing of key presses and releases for every key to the instrument.

Neuroimaging data were acquired on a 3-T Siemens Magnetom Trio scanner with a 32-receive channel head coil. Functional data were acquired using echo-planar imaging pulse sequence in an Interleaved Silent Steady-State (ISSS) paradigm [60], where 5 TRs (7.75s) of image acquisition were followed by 4 ‘silent’ TRs (6.2s) when the auditory stimuli were presented (multi-band acceleration factor 3, repetition time [TR] = 1550 m s, echo time [TE] = 30.4 m s, flip angle [FA] = 56°). We acquired 60 slices in transverse plane orientation with an isotropic voxel size of 2.5 × 2.5 × 2.5 mm. For estimating magnetic field inhomogeneities, we additionally acquired two spin-echo EPIs with an inverted phase-encoding direction. An anatomical T1-weighted scan was acquired at the end of the scanning session using a magnetization-prepared rapid gradient-echo sequence (MPRAGE) with a voxel size of 1× 1 × 1 mm isotropic (field of view = 256 × 176 × 256 mm [A-P; *R*-L; F–H]) in sagittal orientation. For Task I, we acquired a total of 325 vol per participant, and for Tasks II, a total of 165 vol per participant.

At the subject level, fMRI data were preprocessed using a standard fMRIPrep pipeline, including filedmap correction and excluding slice-time correction because of sparse-sampling acquisition [fMRIPrep 21.0.0 [63,64] RRID:SCR_016216, which is based on Nipype 1.6.1 [65,66]; RRID:SCR_002502]. Details of the preprocessing steps can be found in the supplement S7. Subjects were excluded due to excessive motion if 10 or more volumes exceeded the framewise displacement threshold of a single voxel dimension (2.5 mm). Additionally, since the movement of participants might have varied between the experimental conditions, we analyzed the differences in framewise displacement between them (Supplement S10).

The preprocessed functional files were then smoothed with a 6 mm FWHM Gaussian kernel within SPM12 (Wellcome Trust Centre for Neuroimaging, University College, London, UK, http://www.fil.ion.ucl.ac.uk/spm/software/spm12) running on MATLAB 2019b (Mathworks, http://www.mathworks.com).

In the spirit of Open Science, we provide a set of tools for the future studies of musicians within the fMRI environment, including our experimental procedures and the software solutions used (https://osf.io/4rkxu/).

#### 6.3.5 Task I - playing music with naturalistic stimuli

The purpose of this task was to demonstrate the keyboard usage in an MRI setting to study playing naturalistic musical stimuli. Eight musical pieces were selected by an expert from The Chopin University of Music, Warsaw, Poland (AW). The stimuli consisted of a mix of folk, classical and popular melodies (Table 5) of a varying degree of difficulty and were adapted to the capabilities of the keyboard: its scale (the C3–C5 register) and possible ways of articulation. Equally important is the fact that the participants remain in a supine position during scanning with MRI, which precludes the use of technically more complicated musical pieces. However, despite the simplicity of the pieces, forced by the research conditions, there are various challenges of the musical material, also in terms of the texture of the pieces. Two-hand melodies were used in the study, with one of them (most often the right) acting as a melody and the other as an accompaniment (block or broken chords), including examples that use non-imitative polyphonic texture (M03 & M04). The musical pieces have different keys (C major, F major, E minor, F minor, G minor), which requires quick orientation in the specific key layout for each key. Each participant received the score for the musical pieces approximately one week before the scanning session and was instructed to learn to play them from memory, paying attention to the register and the fingering.

**Table 5:** Overview of the musical pieces played by the musicians group.

No	Composer/source	Title (original title)
M01	American holiday song	*Jingle bells*
M02	Y. Tiersen	*Amélie - Nursery Rhyme from Another Summer (Amelie - Comptine d'un autre été)*
M03	Kuyavian folk song	*Red Apple (Czerwone Jabłuszko)*
M04	Ch. Petzold^a^	*Minuet in G Minor from the Notebooks for Anna Magdalena Bach*
M05	Masovian folk song	*Here Comes Maciek (Idzie Maciek)*
M06	A. Dvořák	*Merry-go-round (Karussel)*
M07	R. Schumann	*The Merry Peasant (Fröhlicher Landman)*
M08	F. Chopin	*Lithuanian Song (Piosnka Litewska)*

a Formerly attributed to J.S. Bach.

#### 6.3.6 The task consisted of two conditions (Fig.S9.1a)

- *Listen*: a cue with an ear and notes symbol was presented. The participants heard a fragment of one of the melodies that they were asked to practise at home. They were asked to recognise the fragment and refrain from pressing any keys during the *listen* condition and the scanning time that followed immediately afterwards when a fixation cross was presented. Depending on the following playback block, the heard fragment was played either using both hands or the right hand only.
- *Playback*: a cue with a keyboard symbol was presented with a single dot on the right side of the keyboard for the right hand or two dots on both sides of the keyboard for both hands. The participants were asked to play exactly the fragment that they heard in the *listen* condition directly preceding the current trial, using the hands indicated by the dots. The participants were asked to continue and finish playing the current fragment even if they made a mistake or the time ran out and the scanning time started, recognised by scanner noise and a fixation cross cue.

The conditions were alternating in such a way that after every *listen* condition came a corresponding playback condition. Two easily identifiable excerpts were selected for each musical piece and presented once for the right hand, and once for both hands. Those excerpts were always presented consecutively one after the other (*listen*1 - *playback*1 - *listen*2 - *playback*2), in the same order as they appear in the musical piece and under the same hand condition (right hand only or both hands). E.g., if the participant heard the first excerpt from melody M03, after the playback condition the next excerpt would also come from melody M03 and would naturally occur later in the musical piece than the first excerpt. The order of the melodies was semi-randomised and counterbalanced across participants.

Behavioural data acquired during scanning encompassed the timing of key presses and releases for every key to the instrument. Out of all events across all participants (n = 10,207) constituted by a key press and release, we have found 11 events (0.11%) with missing key press time and 12 trials (0.12%) with missing key release time. We removed those events from further analyses. The participants' performance was scored using a comparison to an errorless performance. For melodic performance, keypresses were encoded as capital letters of the Latin alphabet, such that each key was represented by a single unique character (key0 = C3 = A, key1 = C3# = B, key2 = D3 = C, …, key24 = C5=Y, Table 6). The musical score was encoded similarly. Since the experimental stimuli in *Task I* employed naturalistic polyphonic music, a space character was used to reflect the succession of sounds (single notes or simultaneous polyphonic sound structures). Space-separated single characters represented single (consecutive) sounds of a melody, while multiple characters not separated by spaces represented groups of multiple sounds occurring simultaneously in a musical piece. The characters were sorted alphabetically within a single space-separated group for the musical score, and in preprocessing in the transcription of participants' performances (Fig.S9.2a&b). We assumed the simultaneity of keypresses when the time difference between the logged presses was under 30 m s [67]. The encoded actual and the errorless performance were compared using Levenshtein ratio [36]. This method compares two strings of text letter-by-letter and calculates a distance between them based on the number of errors in relation to their joint length, i.e. the ratio 1-(L_distance_/L_sum_), where L_distance_ is the number of insertions, omissions and substitutions between performed trial and the reference, and L_sum_ is the sum of the lengths, in characters, of both the performed trial and the reference. The resulting score ranges from 0 (no similarity to the errorless performance) to 1 (perfect similarity to the errorless performance). A validation of this metric can be found in the supplement (S4). Using two-way repeated-measures analysis of variance (ANOVA) we tested whether there was a statistically significant difference in similarity ratios among participants in melodies and hand-condition (supplement S5). Bonferroni correction for multiple comparisons was applied in post-hoc analyses. Since the Levenshtein ratio is a value between 0 and 1, there was a suspicion that the performance data would not meet ANOVA's normality assumption. We checked whether the departure from normality of our data would affect the F statistic using skewness and kurtosis limits of 2.31 and 8, respectively [38].

**Table 6:** The mapping of keys to sounds during the experiment and to characters for data analysis.

Key	0	1	2	3	4	5	6	7	8	9	10	11	
Sound	C3	C#3	D3	D#3	E3	F3	F#3	G3	G#3	A3	A#3	B3	
Character	A	B	C	D	E	F	G	H	I	J	K	L	

Key	12	13	14	15	16	17	18	19	20	21	22	23	24

Sound	C4	C#4	D4	D#4	E4	F4	F#4	G4	G#4	A4	A#4	B4	C5
Character	M	N	O	P	Q	R	S	T	U	V	W	X	Y

For rhythmic performance, we applied rhythm-reproduction tapping-PROMS analysis method, for comparing complex rhythmic measures [37]. In short, this method converts timestamps of key presses into proportions of the whole performance, starting from the first key press (e.g. 4 quarter notes would be each expected to occur in 1/3rd of the total time between the first and the last note). The absolute measure of the difference between the measured and expected proportions are then related to the duration of the expected interval between the consecutive keypresses. Thus, the obtained score represents the percentage deviance of each keystroke related to the expected timing and note duration; therefore, the closer the score was to zero, the more similar the performance was to the expected timing. Because this method compares each keypress with a reference, only performances where the total number of keypresses is equal to the expected number of notes are eligible for analysis. As a large number of trials were removed in this manner, repeated-measures ANOVA could not be performed; a regular two-sample *t*-test was used instead to compare rhythmic accuracy of playing with the right hand with playing with both hands. Additionally, because the stimuli contained simultaneous keypresses, only the first keypress from each group of simultaneously played notes (Δt≤30 m s) was considered in the analysis.

For the fMRI data, in the first-level model, the listen (right hand), listen (both hands), playback (right hand) and playback (both hands) conditions were modelled separately, resulting in 4 factor regressors (2 listen and 2 playback). The regressors were constituted of boxcar functions starting at the beginning of the trials and lasting for trial duration. The evoked hemodynamic response to the *listen* and *playback* blocks was modelled as boxcars convolved with a hemodynamic response function (HRF), with all trials included, regardless of their performance scores. A default SPM12 hemodynamic response function was used. Statistical parametric maps were obtained for all 4 conditions.

At the group level, a full factorial 2 × 2 model was constructed using *listen* vs *playback* as one factor and right-hand vs both hands as the other factor. Constructed contrasts included *listen* > *playback, playback* > *listen*, and *playback* (both hands) > *playback* (right hand). A voxel-wise height threshold of p < 0.001 (uncorrected) combined with a cluster-level extent threshold of p < 0.05 (FWE corrected) was applied. The resulting statistical parametric map was exported from SPM and visualised using nilearn in python.

#### 6.3.7 Task II - playback with altered auditory feedback

The aim of this task was to investigate the processing of auditory incongruence in musicians, similarly to what happens when an error is made during musical performance. To simulate errors in a consistent and repeatable manner, we employed an altered auditory feedback (AAF) paradigm, which has previously been used to study auditory incongruence in musicians [25,31]. However, we limited the auditory feedback alteration to a single key in half of the task trials. Eight scales were selected that could be comfortably played in the keyboard's register with the right hand (keys: F major, F# minor, G major, G minor, A minor, Bb major, B minor, C major). All the minor scales were natural minor scales. The scales were played ascending and then descending, resulting in expected 16 key presses per each scale.

The task consisted of two conditions (FigS9.1b).

- *Listen*: a cue with an ear and notes symbol was presented, along with the tonic and the mode, using notation standard to the Polish setting (e.g.: *C*-dur^3^), was presented. The participants heard the heptatonic scale corresponding to the cue, played forwards and backwards. Participants were asked to refrain from pressing any keys during the *listen* condition and the scanning time that followed immediately afterwards when a fixation cross was presented.
- *Playback*: The participants were asked to repeat the scale that they just heard. In half of the trials, the auditory feedback on one of the keys was manipulated such that the heard pitch differed from the target key pitch by a halftone or a whole tone. The participants were instructed that sometimes, what they hear might be different from what they expect, with no further explanation. They were also asked to keep playing no matter what they heard. If the time allotted for the playback condition ran out, and the scanning time of the ISSS paradigm started (recognised by an increase in the scanner noise and a fixation cross cue, the participants were instructed to finish playing the scale.

Similarly to the playback task, the participants were alternating the listen and the playback trials. Each scale appeared three times, of which one or two playback trials had altered auditory feedback. The order of the trials was semi-randomised and counterbalanced across participants.

The participants’ keypresses were logged, transcribed and compared to an errorless performance (see *Task I* for details), however, the space character was not used to separate notes, as no structures of simultaneous sounds were expected (Fig.S9.2c&d). To check whether AAF impacted the overall performance, we compared auditory feedback conditions (altered vs standard) using a one-way repeated-measures ANOVA. Additionally, we checked whether the keypresses were delayed after the key with the altered auditory feedback was pressed, in comparison to the previous keys. For each scale, we averaged the time between keypresses for the three keys preceding the first target key with altered auditory feedback, and the three keys after this target key. These means were then compared using a one-way repeated-measures ANOVA.

For neuroimaging data, in the first-level analysis, the *listen* condition was divided into trials corresponding to the playback trials with standard and with altered auditory feedback, resulting in four factor regressors (2 *listen* and 2 *playback*). The regressors were constituted of boxcar functions starting at the beginning of the trials and lasting for trial duration. The evoked hemodynamic response to the *listen* and *playback* blocks was modelled as boxcars convolved with a hemodynamic response function (HRF), with all trials included, regardless of their performance scores. The boxcar function was convolved with a default SPM12 hemodynamic response function. Statistical parametric maps were obtained for all 4 conditions.

At the group level, a full factorial 2 × 2 model was constructed using *listen* vs *playback* as one factor and standard vs altered auditory feedback as the other factor. A playback (altered auditory feedback) - playback (standard feedback) contrast was constructed using one-sample t-tests. A voxel-wise height threshold of p < 0.001 (uncorrected) combined with a cluster-level extent threshold of p < 0.05 (FWE corrected) equal to 133 voxels was applied. The resulting statistical parametric map was exported from SPM and visualised using nilearn in python.

### 6.4 Quantification and statistical analyses

#### 6.4.1 Sample size

Previous studies of MRI-compatible keyboards had a sample size between 16 [14] and 22 [12] pianists, while studies on altered auditory feedback had a sample size of 15 [31] and 20 [25] participants. Thus, we decided to recruit 20 participants.

Throughout the study, we use the following notions of sample sizes.

1. Number of participants (N = 20 for Task I and N = 16 for Task II)
2. Task I: Performance of melodies (n = 640 performances across 20 participants, 2 hand conditions and 8 melodies) and rhythmic performance (N = 153, of which 3 outliers)
3. Task II: The average performance of scales (n = 16 participants, 2 conditions of auditory feedback), the mean time between keypresses (n = 16, before and after AAF key)

#### 6.4.2 Statistical analyses

Both tasks: Statistical analysis of the behavioural data was performed using the Pingouin v0.5.1 package [68] in Python v. 3.10 [69].

Task I: A difference in participants' melodic performance (Levenshtein's ratio) in melodies and hand-condition was tested using two-way repeated-measures analysis of variance (ANOVA). Bonferroni correction for multiple comparisons was applied in post-hoc analyses. For rhythmic performance, because certain melodies and hand condition combinations yielded no eligible trials, repeated-measures ANOVA could not be performed (Supplement S6); a regular two-sample *t*-test was used instead to compare melodies played with the right hand to melodies played with both hands. For each hand condition, outliers ( ≥ 3SD) were removed before the statistical analysis (3 trials total).

Task II: To check whether AAF impacted the overall performance, we compared auditory feedback conditions (altered vs standard) using a one-way repeated-measures ANOVA. The mean times between consecutive keypresses before and after AAF were also compared using a one-way repeated-measures ANOVA.

Both tasks

At the subject level, neuroimaging analyses were performed using a general linear model [70] on a whole-brain level. To correct for sparse acquisition, the first acquired volume was used as a dummy scan to fill in the ‘silence’ gaps, a ‘0’-vector to expand the head movement regressors (translation and rotation in X, Y and Z directions), and an additional regressor was added in the model to indicate which scans were actual scans and which were the dummy scans [23]. The experimental conditions were modelled separately, resulting in 4 factor regressors (2 *listen* and 2 *playback*). The regressors were constituted of boxcar functions starting at the beginning of the trials and lasting for trial duration. The evoked hemodynamic response to the *listen* and *playback* blocks was modelled as boxcars convolved with a hemodynamic response function (HRF), with all trials included, regardless of their performance scores. A default SPM12 hemodynamic response function was used. Statistical parametric maps were obtained for all 4 conditions.

At the group level, both tasks used a full-factorial design. In Task I, one factor corresponded to the *listening* vs *playback* conditions, and the other to the *right hand* vs *both hands* conditions. In Task II, one factor corresponded to the *listening* vs *playback* conditions, and the other to the *altered auditory feedback* vs *standard auditory feedback* conditions. To correct for multiple comparisons, a voxel-wise height threshold of p < 0.001 (uncorrected) combined with a cluster-level extent threshold of p < 0.05 (FWE corrected) was applied.

## Data availability statement

The source data and code used to create the figures Fig. 3, Fig. 5 - behavioural performance of the fMRI tasks, Fig. 4a, Fig. 4ba and b - brain activation in response to the fMRI tasks) as well as thresholded statistical maps from all whole brain group analysis are available in the Open Science Framework project:https://osf.io/4rkxu/. Due to copyright, the musical stimuli will be made available on request.

## Funding

This study was supported by the 10.13039/501100004281National Science Centre grant number 2018/30/E/HS6/00206. AH is supported by the Foundation for Polish Science (FNP).

## Author contribution statement

Alicja M. Olszewska: Conceived and designed the experiments; Performed the experiments; Analyzed and interpreted the data; Wrote the paper.

Dawid Droździel: Conceived and designed the experiments; Performed the experiments.

Maciej Gaca: Analyzed and interpreted the data.

Agnieszka Kulesza: Performed the experiments.

Wojciech Obrębski: Conceived and designed the experiments; Performed the experiments; Analyzed and interpreted the data; Contributed reagents, materials, analysis tools or data; Wrote the paper.

Jakub Kowalewski: Conceived and designed the experiments; Performed the experiments; Analyzed and interpreted the data; Contributed reagents, materials, analysis tools or data.

Agnieszka Widlarz; Artur Marchewka: Conceived and designed the experiments; Contributed reagents, materials, analysis tools or data.

Aleksandra M. Herman: Conceived and designed the experiments; Performed the experiments; Analyzed and interpreted the data.

## Declaration of competing interest

WO and JK own the SMIT-Lab company, which is the manufacturer of the MRI-compatible keyboard featured in this article. Remaining authors declare no conflict of interest.

## Footnotes

3 eng. C major. In the Polish musical notation, major scales are written in capital letters with the suffix ‘-dur’, and minor scales are written in lowercase letters with the suffix ‘-mol’. Sharp notes are denoted either with # or with the suffix ‘is’ (e.g. f# - fis) and flat notes either with ♭ or with the suffix “es” (e.g. g♭- ges).
