# Supplementary material for: Unlocking the musical brain: A proof-of-concept study on playing the piano in MRI scanner with naturalistic stimuli
Source: Heliyon. 2023 Jul 11;9(7):e17877. doi: 10.1016/j.heliyon.2023.e17877 (PMC10368778; doi:10.1016/j.heliyon.2023.e17877)
Supplement: Multimedia component 1 [file mmc1.docx]

## Supplementary material

### S1: Detailed description of the device and its interface:

The keyboard involves a key-mechanism adapted from commercially available MIDI keyboard modified for MRI applications. Metal springs were replaced with specially designed rubber substitutes. The mechanism was attached to the dedicated Printed Circuit Board with the necessary electronics and two electrical, surface contacts for each key. When the key is pushed, those two contacts are closed with a delay relative one to another, which depends on the keypress velocity. The delay is measured by the microcontroller and converted to a value in a range between 0-127 which is a MIDI velocity factor sent with MIDI note. Velocity indicates how hard the key was struck when the note was played, which corresponds to the note's loudness. The velocity-to-loudness converting function implemented in the microcontroller depends on a user/researcher selectable parameter. This way, it is possible to adjust converting function behaviour. This enables one to modify the keyboard's articulative characteristics to mimic another keyboard, for example, a training keyboard used outside of the scanner room. Implemented dynamic sound articulation is similar to solutions utilised in professional keyboards commonly used by musicians. Moreover, the researcher can dynamically change the note number sent by each key, which means that the keys can be "swapped" during the experiment. This feature also allows simple octave shifting for the whole keyboard.

The keyboard is the element of the wireless network of the S2 system by Smit-Lab.  The network is based on IEEE 802.15.1 standard with full duplex communication. Each event from a wireless device is time-stamped by a hardware clock with 1ms accuracy. The events are collected by the network concentrator in Faraday Cage room and then they are transmitted via fibre optic bus outside of the cage to the Control box device (Fig.4b). Fibre optic bus is a part of the local area network (LAN) so each event is available for any computer in the laboratory.  Any authorised computer in the laboratory can also change settings of the MIDI keyboard (such as key swapping). To do that, computers can use the S2 PC application dedicated system (Fig. S1.1), which can change settings but also log every event in it.


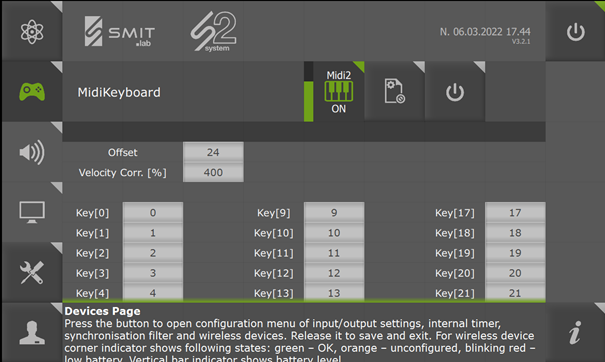


Fig. S1.1. The interface of the S2 system, displaying the keyboard settings. The “Offset” setting shifts the whole register of the keyboard by N tones, which allows for serial shifting of the pitches played by the participant, similarly to (Pfordresher et al. 2014). Velocity Corr. [%] setting adjusts the velocity-to-loudness conversion, changing the keyboard’s articulative characteristics. The numbers for each key represent a pitch assigned to each key, based on the Offset parameter. All of the parameters (the offset, the velocity correction and the pitch-per-key mapping) can be adjusted on-the-fly.

Alternatively, a dedicated plug-in for Presentation software can be used (Fig. S1.2a&b).


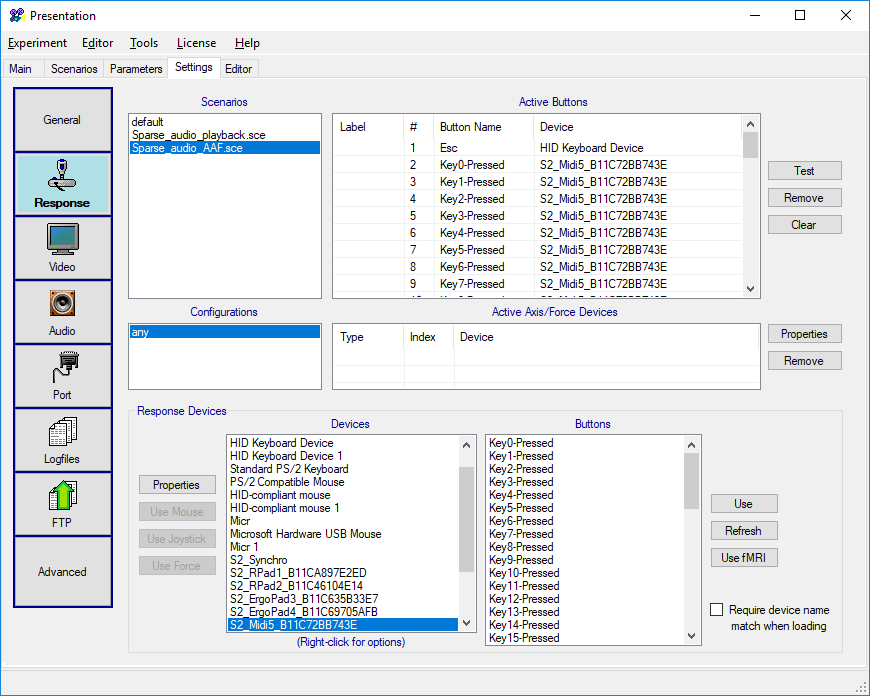


Fig. S1.2a. The interface of the Presentation (Neurobehavioral Systems, Inc., Berkeley, CA, www.neurobs.com) software. Using a dedicated plug-in, events based on keypress and release from the fMRI MIDI keyboard can be logged and used in the experimental procedures just like any other response pad.


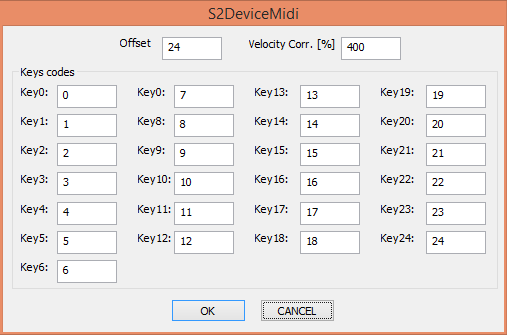


Fig. S1.2b. The interface of the Presentation (Neurobehavioral Systems, Inc., Berkeley, CA, www.neurobs.com) software. Using a dedicated plug-in, the settings of the keyboard can be adjusted also from the level of the experimental software, both via the graphical user interface and in the programmed experimental procedures.

Network protocol uses unicast UDP (User Datagram Protocol) packets for configuration and multicast UDP packets for events. It is a simple, custom protocol available on request to any user, so it is easy to implement the communication between the system and any research software. The PsychoPy plugin is under development and the system is being upgraded to support a lab streaming layer (LSL).23

The control box device is equipped with a configurable USB which can fulfil the USB MIDI 1.0 specification. Thanks to that all events on the LAN are also available via USB bus. In this scenario USB can be plugged to the PC with a software MIDI synthesiser but a better, more reliable way that secures the minimal delay is to connect it to the MIDI sound module instead.

The keyboard solution, with or without the S2 module, is commercially available and ready-to-use as described in the manuscript, without the need for further modifications. At the moment, a dedicated Presentation plugin is included, and a plugin for PsychoPy is being prepared.

### S2: Testing for potential artefacts of the device in the scanner signal

We tested the noise levels in the scanner signal and observed no difference between the setup with the keyboard being on or off, and no frequency spikes related to data transmission (Fig. S2). The 32-channel head-coil used in this study is operating very locally, and we do not expect that a device placed on the subjects’ laps will influence the signal.


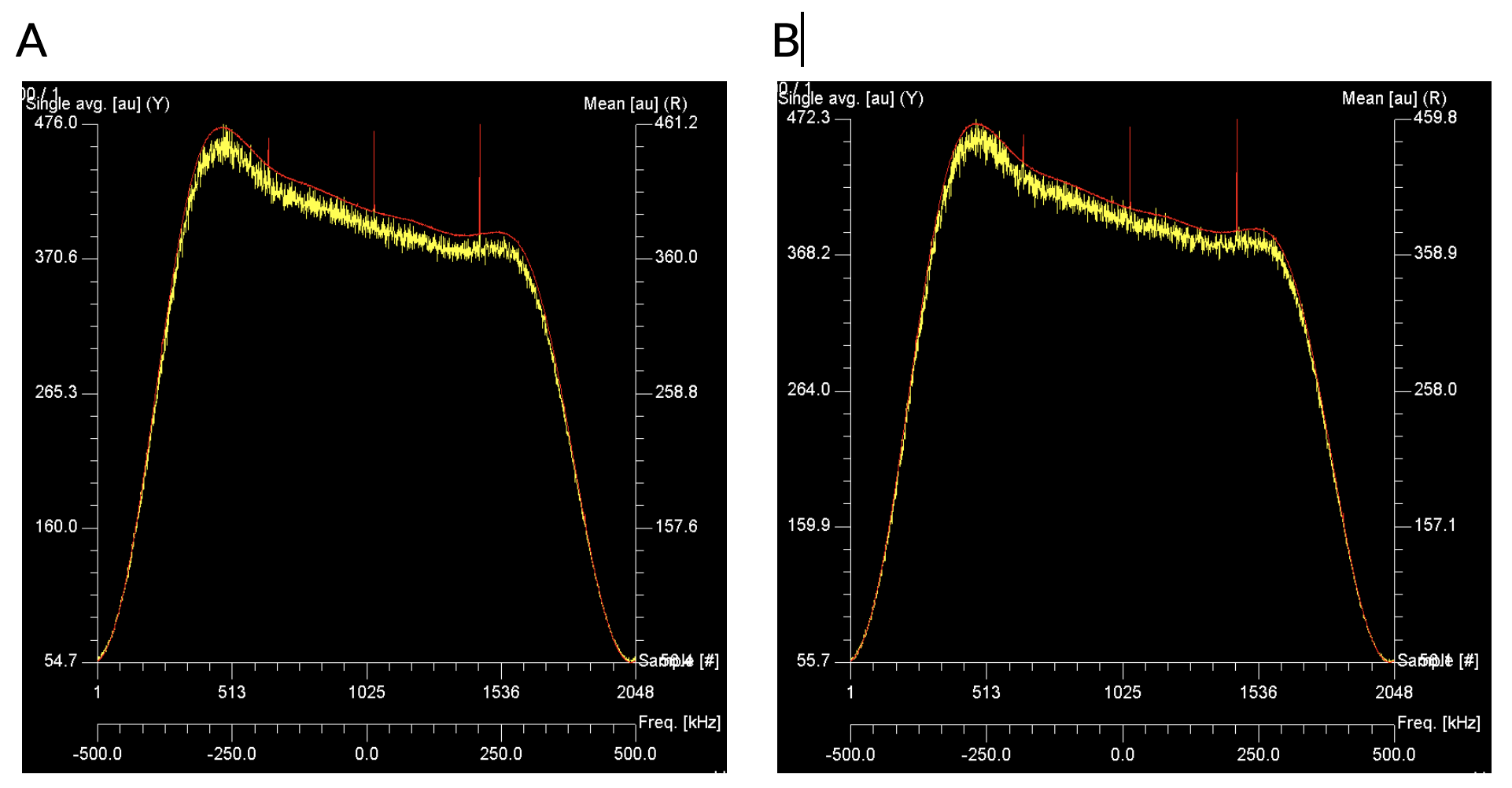


Fig. S2. Noise spectrum with the keyboard being off (A) and on (B) recorded with a 32-channel coil, transmit coil switched off. X axis: tested frequency; Y axis: noise amplitude.

### S3: The breakdown of the device’s latency:

The slowest interface is the radio interface based on the IEEE 802.15.1 specification. The radio link was realised with a CC2564C chip (Texas Instruments) on the Keyboard side and LMX9830 chip (Texas Instrument) on Antenna BOX side. The theoretical speed for Asynchronous Connection-Less (ACL) protocol defined for IEEE 802.15.1 in downlink mode is 57.6 Kbps. For each keypress event, 22 bytes are sent over radio (key number, key status, timestamp, etc.) and, for this frame length, the resulting delay is approximately 3 ms. Another 1 ms delay will be inserted by MIDI transfer so 4 ms is the theoretical minimum delay for the whole system. However, ACL connections have a retransmission mechanism. Retransmission may occur in the event of a packet collision in the air, which may additionally extend the transmission process. In our case, the device is used in an isolated environment (in a Faraday cage), so a collision is unlikely, but the tomograph itself can trigger it during high RF power pulses.

### S4: Validation of the Levenshtein Ratio metric

To give insight into the performance of our index of musical performance (Levenshtein ratio), we compared it to the standard note-by-note scoring method for the experimental data from our experiment (AAF).

In the note-by-note metric the note sequences are compared note-by-note and the score is increased by 1 each time the note in the reference sequence and the actual performance sequence is the same at the very same position, yielding a maximal score of 16 (16 notes in the reference sequence). To directly compare this method to the Levenshtein ratio, we normalised the score by dividing it by 16. The note-by-note method is very sensitive to the position of the first error in the sequence, yielding decreased scores if an error appears early on in the sequence. This means that the same number of errors can be represented by different scores, and the overall score is a combination of the number of errors and their position. The metric we propose, based on the similarity to an errorless performance (Levenshtein ratio i.e. the ratio 1-(L_distance_/L_sum_), where L_distance_ is the number of insertions, omissions and substitutions between performed trial and the reference, and L_sum_ is the sum of the lengths, in characters, of both the performed trial and the reference), is robust in the way that it is not influenced by the position of errors, only their number.


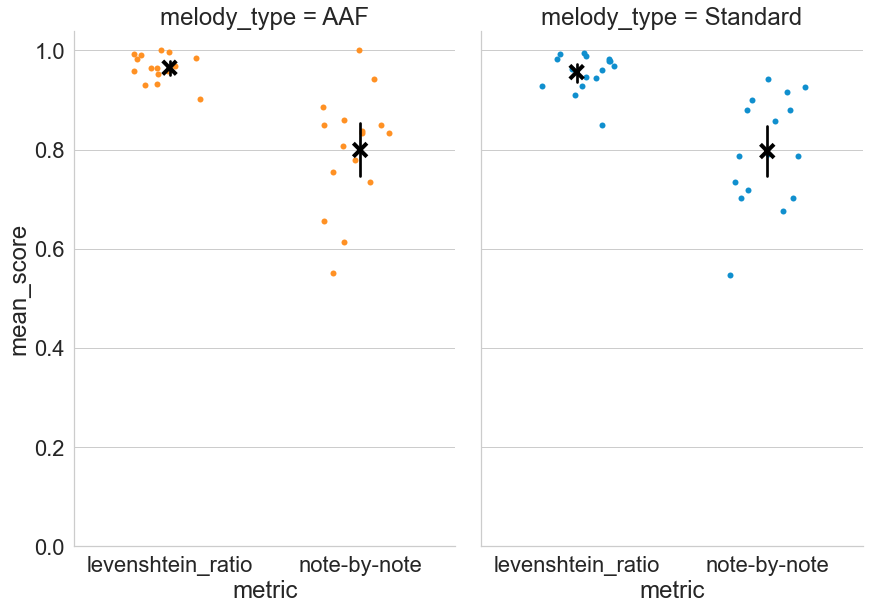


Fig. S4.1. A comparison between the performance metrics in the AAF and the Standard feedback conditions. Each dot represents a mean performance per participant, the X indicates a mean overall performance across participants, and the error bars represent the 95% confidence intervals. A significant difference between metric scores can be observed (AAF: df: 15, F = 39.9, p<0.01; Standaard feedback: df: 15, F:47.1, p<0.01)

In Fig. S4.1 above we present the comparison of the mean Levenshtein ratio metric (left) and the mean Note-by-Note Score (right) methods for the behavioural performance in the AAF task for the AAF and Standard feedback conditions in our participants. The results between the metrics are statistically significant (p<0.01). The Note-by-Note scores are characterised by higher variance and lower mean, because the position of the first error influences the score.

To additionally illustrate the robustness of our metric, we added an “error” by extending the sequences by a single sound (Fig. S4.2). We added the same letter at the beginning or the end of the metric. Thus, the number of errors increases by 1 for each performance, but the position of the additional “error” is either on the first or the last character in the sequence. In the case of our metric (Levenshtein ratio), the position of the introduced error does not affect the score, while in the case of the note-by-note metric, it affects the score drastically.

a


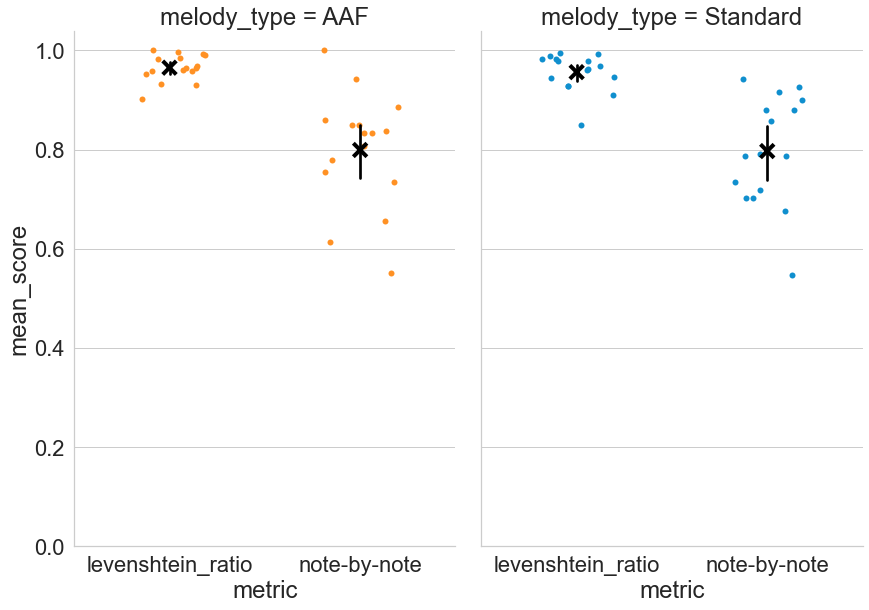


b


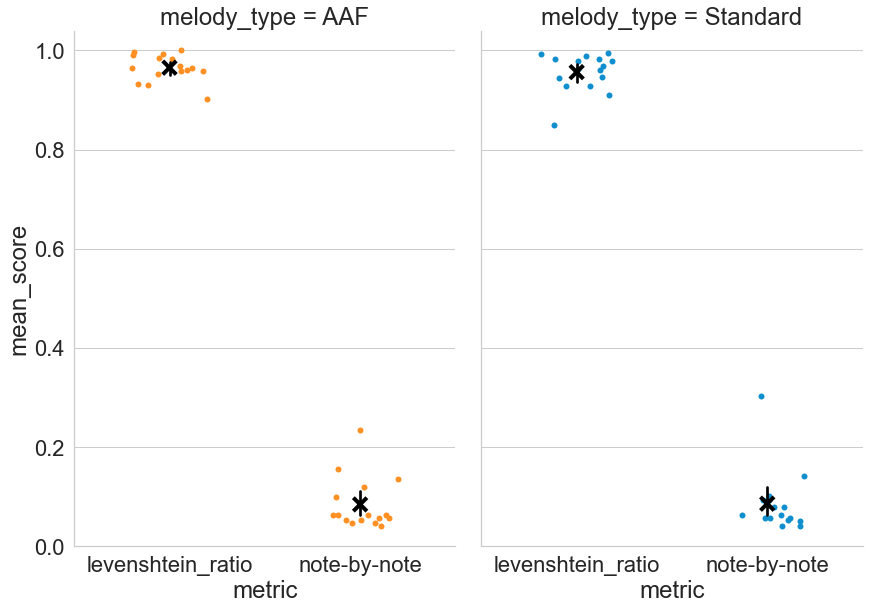


Fig. S4.2. The introduction of an artificial “error” at the beginning of a sequence (a) or end of a sequence (b) drastically changes the note-by-note metric, despite the number of errors being the same. The Levenshtein ratio metric is robust and depends on the number of errors, not their positions.

### S5: Post-hoc analyses for the behavioural data of experiment I:

|  | A | B | T-value | dof | p (bonf.corr) |
| --- | --- | --- | --- | --- | --- |
|  | M01 | M02 | -0.270129 | 19 | 1.000000 |
|  | M01 | M03 | -0.405286 | 19 | 1.000000 |
|  | M01 | M04 | 0.191974 | 19 | 1.000000 |
|  | M01 | M05 | 0.174238 | 19 | 1.000000 |
|  | M01 | M06 | -0.849540 | 19 | 1.000000 |
|  | **M01** | **M07** | **5.656999** | **19** | **0.000525** |
|  | M01 | M08 | 3.504989 | 19 | 0.066315 |
|  | M02 | M03 | -0.288171 | 19 | 1.000000 |
|  | M02 | M04 | 0.612709 | 19 | 1.000000 |
|  | M02 | M05 | 0.404369 | 19 | 1.000000 |
|  | M02 | M06 | -0.451929 | 19 | 1.000000 |
|  | **M02** | **M07** | **5.701237** | **19** | **0.000477** |
|  | M02 | M08 | 3.007922 | 19 | 0.202543 |
|  | M03 | M04 | 0.653911 | 19 | 1.000000 |
|  | M03 | M05 | 0.601155 | 19 | 1.000000 |
|  | M03 | M06 | -0.179026 | 19 | 1.000000 |
|  | **M03** | **M07** | **6.080436** | **19** | **0.000212** |
|  | M03 | M08 | 3.163578 | 19 | 0.143216 |
|  | M04 | M05 | -0.022739 | 19 | 1.000000 |
|  | M04 | M06 | -0.929159 | 19 | 1.000000 |
|  | **M04** | **M07** | **5.140166** | **19** | **0.001629** |
|  | M04 | M08 | 2.832846 | 19 | 0.297744 |
|  | M05 | M06 | -1.228624 | 19 | 1.000000 |
|  | **M05** | **M07** | **4.574700** | **19** | **0.005796** |
|  | **M05** | **M08** | **4.490657** | **19** | **0.007012** |
|  | **M06** | **M07** | **5.869521** | **19** | **0.000332** |
|  | **M06** | **M08** | **5.128088** | **19** | **0.001674** |
|  | M07 | M08 | -2.877681 | 19 | 0.269909 |
|  | **both_hands** | **right_hand** | **-4.956362** | **19** | **0.000088 (uncorr.)** |
| **M01** | **both_hands** | **right_hand** | **-4.358459** | **19** | **0.002706** |
| M02 | both_hands | right_hand | -2.054597 | 19 | 0.431469 |
| M03 | both_hands | right_hand | -1.285162 | 19 | 1.000000 |
| M04 | both_hands | right_hand | 0.057752 | 19 | 1.000000 |
| **M05** | **both_hands** | **right_hand** | **-4.537884** | **19** | **0.001800** |
| **M06** | **both_hands** | **right_hand** | **-6.237283** | **19** | **0.000044** |
| M07 | both_hands | right_hand | 1.371811 | 19 | 1.000000 |
| **M08** | **both_hands** | **right_hand** | **-4.420248** | **19** | **0.002351** |

### S6: Participants’ performances tapping-PROMS eligibility

Table S6. The number of performances which meet the eligibility criterion for the rhythmic analysis according to the rhythm-reproduction tapping-PROMS. RH=right hand only, BH=both hands.

| Melody | played with | eligible for analysis | ineligible for analysis |
| --- | --- | --- | --- |
| MO1 | RH | 20 | 20 |
|  | BH | 8 | 32 |
| M02 | RH | 23 | 17 |
|  | BH | 10 | 30 |
| M03 | RH | 22 | 18 |
|  | BH | 16 | 24 |
| M04 | RH | 6 | 34 |
|  | BH | 8 | 32 |
| M05 | RH | 14 | 26 |
|  | BH | 7 | 33 |
| M06 | RH | 2 | 38 |
|  | BH | - | 40 |
| M07 | RH | - | 40 |
|  | BH | 5 | 35 |
| M08 | RH | 5 | 35 |
|  | BH | 7 | 33 |


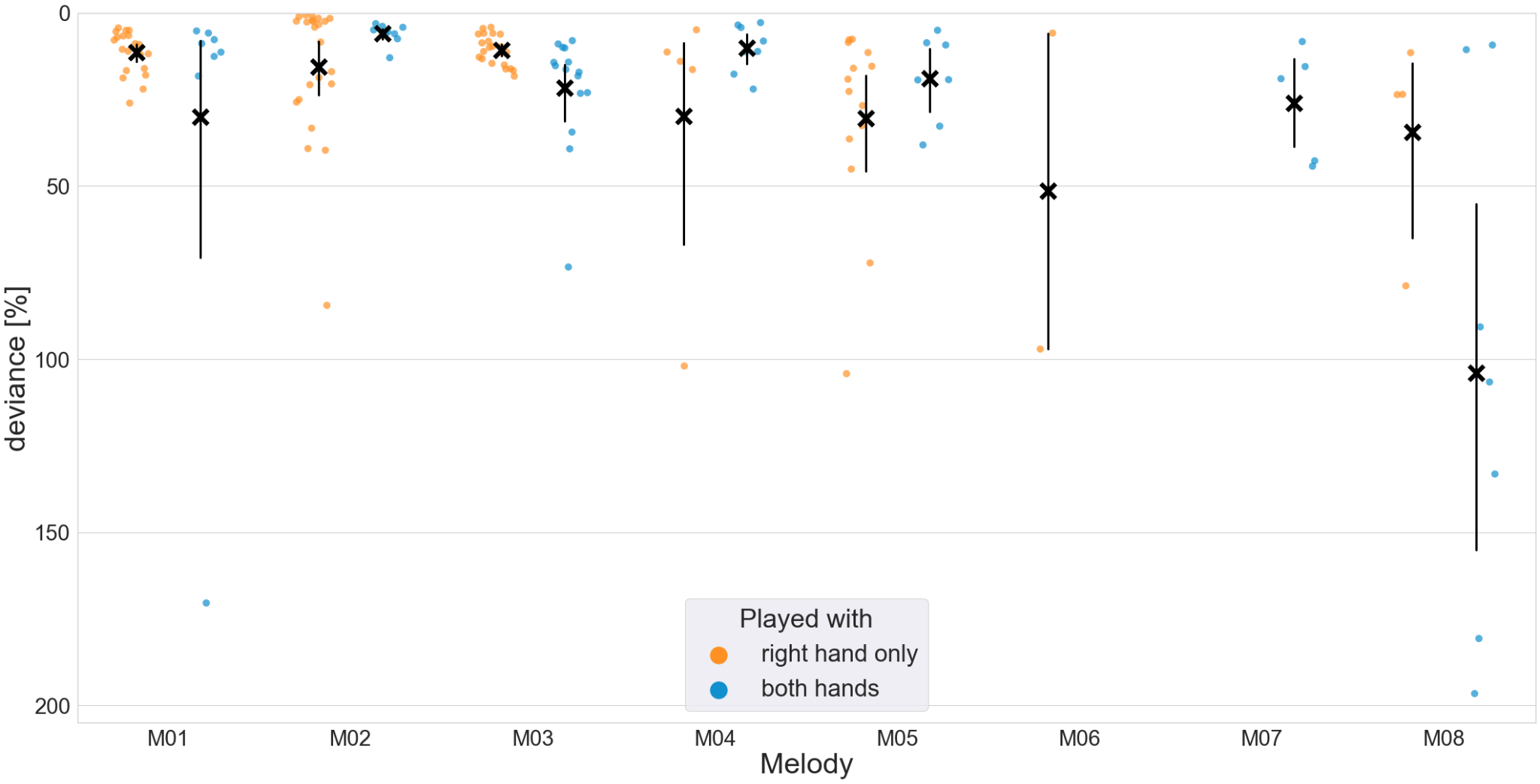


Fig. S6. The rhythmic performance between melodies and hand-conditions. ✖ indicates the mean performance score per melody, bars represent the Standard Errors of the Mean (SEM). Dots represent individual participants’ performance scores.

### S7: The output of the fMRIPrep software

Results included in this manuscript come from preprocessing performed using fMRIPrep 21.0.0 (Esteban, Markiewicz, et al. (2018); Esteban, Blair, et al. (2018); RRID:SCR_016216), which is based on Nipype 1.6.1 (K. Gorgolewski et al. (2011); K. J. Gorgolewski et al. (2018); RRID:SCR_002502).

Preprocessing of B0 inhomogeneity mappings

A total of 1 fieldmaps were found available within the input BIDS structure for this particular subject. A B0-nonuniformity map (or fieldmap) was estimated based on two (or more) echo-planar imaging (EPI) references with topup (Andersson, Skare, and Ashburner (2003); FSL 6.0.5.1:57b01774).

Anatomical data preprocessing

A total of 1 T1-weighted (T1w) images were found within the input BIDS dataset.The T1-weighted (T1w) image was corrected for intensity non-uniformity (INU) with N4BiasFieldCorrection (Tustison et al. 2010), distributed with ANTs 2.3.3 (Avants et al. 2008, RRID:SCR_004757), and used as T1w-reference throughout the workflow. The T1w-reference was then skull-stripped with a Nipype implementation of the antsBrainExtraction.sh workflow (from ANTs), using OASIS30ANTs as target template. Brain tissue segmentation of cerebrospinal fluid (CSF), white-matter (WM) and gray-matter (GM) was performed on the brain-extracted T1w using fast (FSL 6.0.5.1:57b01774, RRID:SCR_002823, Zhang, Brady, and Smith 2001). Volume-based spatial normalization to one standard space (MNI152NLin2009cAsym) was performed through nonlinear registration with antsRegistration (ANTs 2.3.3), using brain-extracted versions of both T1w reference and the T1w template. The following template was selected for spatial normalization: ICBM 152 Nonlinear Asymmetrical template version 2009c [Fonov et al. (2009), RRID:SCR_008796; TemplateFlow ID: MNI152NLin2009cAsym].

Functional data preprocessing

For each of the 1 BOLD runs found per subject (across all tasks and sessions), the following preprocessing was performed. First, a reference volume and its skull-stripped version were generated using a custom methodology of fMRIPrep. Head-motion parameters with respect to the BOLD reference (transformation matrices, and six corresponding rotation and translation parameters) are estimated before any spatiotemporal filtering using mcflirt (FSL 6.0.5.1:57b01774, Jenkinson et al. 2002). The estimated fieldmap was then aligned with rigid-registration to the target EPI (echo-planar imaging) reference run. The field coefficients were mapped on to the reference EPI using the transform. The BOLD reference was then co-registered to the T1w reference using mri_coreg (FreeSurfer) followed by flirt (FSL 6.0.5.1:57b01774, Jenkinson and Smith 2001) with the boundary-based registration (Greve and Fischl 2009) cost-function. Co-registration was configured with six degrees of freedom. Several confounding time-series were calculated based on the preprocessed BOLD: framewise displacement (FD), DVARS and three region-wise global signals. FD was computed using two formulations following Power (absolute sum of relative motions, Power et al. (2014)) and Jenkinson (relative root mean square displacement between affines, Jenkinson et al. (2002)). FD and DVARS are calculated for each functional run, both using their implementations in Nipype (following the definitions by Power et al. 2014). The three global signals are extracted within the CSF, the WM, and the whole-brain masks. Additionally, a set of physiological regressors were extracted to allow for component-based noise correction (CompCor, Behzadi et al. 2007). Principal components are estimated after high-pass filtering the preprocessed BOLD time-series (using a discrete cosine filter with 128s cut-off) for the two CompCor variants: temporal (tCompCor) and anatomical (aCompCor). tCompCor components are then calculated from the top 2% variable voxels within the brain mask. For aCompCor, three probabilistic masks (CSF, WM and combined CSF+WM) are generated in anatomical space. The implementation differs from that of Behzadi et al. in that instead of eroding the masks by 2 pixels on BOLD space, the aCompCor masks are subtracted a mask of pixels that likely contain a volume fraction of GM. This mask is obtained by thresholding the corresponding partial volume map at 0.05, and it ensures components are not extracted from voxels containing a minimal fraction of GM. Finally, these masks are resampled into BOLD space and binarized by thresholding at 0.99 (as in the original implementation). Components are also calculated separately within the WM and CSF masks. For each CompCor decomposition, the k components with the largest singular values are retained, such that the retained components’ time series are sufficient to explain 50 percent of variance across the nuisance mask (CSF, WM, combined, or temporal). The remaining components are dropped from consideration. The head-motion estimates calculated in the correction step were also placed within the corresponding confounds file. The confound time series derived from head motion estimates and global signals were expanded with the inclusion of temporal derivatives and quadratic terms for each (Satterthwaite et al. 2013). Frames that exceeded a threshold of 0.5 mm FD or 1.5 standardised DVARS were annotated as motion outliers. The BOLD time-series were resampled into standard space, generating a preprocessed BOLD run in MNI152NLin2009cAsym space. First, a reference volume and its skull-stripped version were generated using a custom methodology of fMRIPrep. All resamplings can be performed with a single interpolation step by composing all the pertinent transformations (i.e. head-motion transform matrices, susceptibility distortion correction when available, and co-registrations to anatomical and output spaces). Gridded (volumetric) resamplings were performed using antsApplyTransforms (ANTs), configured with Lanczos interpolation to minimize the smoothing effects of other kernels (Lanczos 1964). Non-gridded (surface) resamplings were performed using mri_vol2surf (FreeSurfer).

Many internal operations of fMRIPrep use Nilearn 0.8.1 (Abraham et al. 2014, RRID:SCR_001362), mostly within the functional processing workflow. For more details of the pipeline, see the section corresponding to workflows in fMRIPrep’s documentation.

Copyright Waiver

The above boilerplate text was automatically generated by fMRIPrep with the express intention that users should copy and paste this text into their manuscripts unchanged. It is released under the CC0 license.

References

Abraham, Alexandre, Fabian Pedregosa, Michael Eickenberg, Philippe Gervais, Andreas Mueller, Jean Kossaifi, Alexandre Gramfort, Bertrand Thirion, and Gael Varoquaux. 2014. “Machine Learning for Neuroimaging with Scikit-Learn.” Frontiers in Neuroinformatics 8. https://doi.org/10.3389/fninf.2014.00014.

Andersson, Jesper L. R., Stefan Skare, and John Ashburner. 2003. “How to Correct Susceptibility Distortions in Spin-Echo Echo-Planar Images: Application to Diffusion Tensor Imaging.” NeuroImage 20 (2): 870–88. https://doi.org/10.1016/S1053-8119(03)00336-7.

Avants, B. B., C. L. Epstein, M. Grossman, and J. C. Gee. 2008. “Symmetric Diffeomorphic Image Registration with Cross-Correlation: Evaluating Automated Labeling of Elderly and Neurodegenerative Brain.” Medical Image Analysis 12 (1): 26–41. https://doi.org/10.1016/j.media.2007.06.004.

Behzadi, Yashar, Khaled Restom, Joy Liau, and Thomas T. Liu. 2007. “A Component Based Noise Correction Method (CompCor) for BOLD and Perfusion Based fMRI.” NeuroImage 37 (1): 90–101. https://doi.org/10.1016/j.neuroimage.2007.04.042.

Esteban, Oscar, Ross Blair, Christopher J. Markiewicz, Shoshana L. Berleant, Craig Moodie, Feilong Ma, Ayse Ilkay Isik, et al. 2018. “fMRIPrep.” Software. https://doi.org/10.5281/zenodo.852659.

Esteban, Oscar, Christopher Markiewicz, Ross W Blair, Craig Moodie, Ayse Ilkay Isik, Asier Erramuzpe Aliaga, James Kent, et al. 2018. “fMRIPrep: A Robust Preprocessing Pipeline for Functional MRI.” Nature Methods. https://doi.org/10.1038/s41592-018-0235-4.

Fonov, VS, AC Evans, RC McKinstry, CR Almli, and DL Collins. 2009. “Unbiased Nonlinear Average Age-Appropriate Brain Templates from Birth to Adulthood.” NeuroImage 47, Supplement 1: S102. https://doi.org/10.1016/S1053-8119(09)70884-5.

Gorgolewski, K., C. D. Burns, C. Madison, D. Clark, Y. O. Halchenko, M. L. Waskom, and S. Ghosh. 2011. “Nipype: A Flexible, Lightweight and Extensible Neuroimaging Data Processing Framework in Python.” Frontiers in Neuroinformatics 5: 13. https://doi.org/10.3389/fninf.2011.00013.

Gorgolewski, Krzysztof J., Oscar Esteban, Christopher J. Markiewicz, Erik Ziegler, David Gage Ellis, Michael Philipp Notter, Dorota Jarecka, et al. 2018. “Nipype.” Software. https://doi.org/10.5281/zenodo.596855.

Greve, Douglas N, and Bruce Fischl. 2009. “Accurate and Robust Brain Image Alignment Using Boundary-Based Registration.” NeuroImage 48 (1): 63–72. https://doi.org/10.1016/j.neuroimage.2009.06.060.

Jenkinson, Mark, Peter Bannister, Michael Brady, and Stephen Smith. 2002. “Improved Optimization for the Robust and Accurate Linear Registration and Motion Correction of Brain Images.” NeuroImage 17 (2): 825–41. https://doi.org/10.1006/nimg.2002.1132.

Jenkinson, Mark, and Stephen Smith. 2001. “A Global Optimisation Method for Robust Affine Registration of Brain Images.” Medical Image Analysis 5 (2): 143–56. https://doi.org/10.1016/S1361-8415(01)00036-6.

Lanczos, C. 1964. “Evaluation of Noisy Data.” Journal of the Society for Industrial and Applied Mathematics Series B Numerical Analysis 1 (1): 76–85. https://doi.org/10.1137/0701007.

Power, Jonathan D., Anish Mitra, Timothy O. Laumann, Abraham Z. Snyder, Bradley L. Schlaggar, and Steven E. Petersen. 2014. “Methods to Detect, Characterize, and Remove Motion Artifact in Resting State fMRI.” NeuroImage 84 (Supplement C): 320–41. https://doi.org/10.1016/j.neuroimage.2013.08.048.

Satterthwaite, Theodore D., Mark A. Elliott, Raphael T. Gerraty, Kosha Ruparel, James Loughead, Monica E. Calkins, Simon B. Eickhoff, et al. 2013. “An improved framework for confound regression and filtering for control of motion artifact in the preprocessing of resting-state functional connectivity data.” NeuroImage 64 (1): 240–56. https://doi.org/10.1016/j.neuroimage.2012.08.052.

Tustison, N. J., B. B. Avants, P. A. Cook, Y. Zheng, A. Egan, P. A. Yushkevich, and J. C. Gee. 2010. “N4itk: Improved N3 Bias Correction.” IEEE Transactions on Medical Imaging 29 (6): 1310–20. https://doi.org/10.1109/TMI.2010.2046908.

Zhang, Y., M. Brady, and S. Smith. 2001. “Segmentation of Brain MR Images Through a Hidden Markov Random Field Model and the Expectation-Maximization Algorithm.” IEEE Transactions on Medical Imaging 20 (1): 45–57. https://doi.org/10.1109/42.906424.

### S8: Unthresholded statistical parametric maps

a) *listen* > *playback*


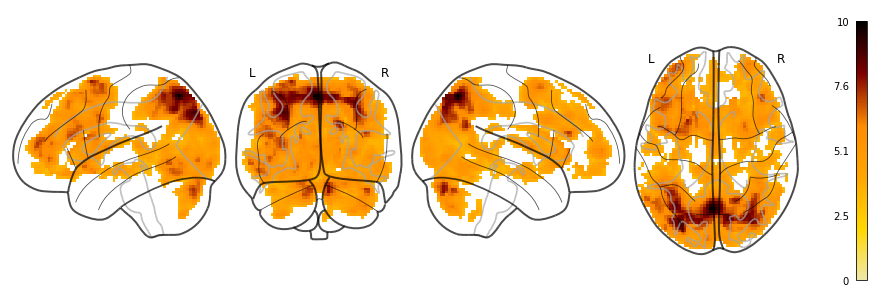


b) *playback* > *listen*


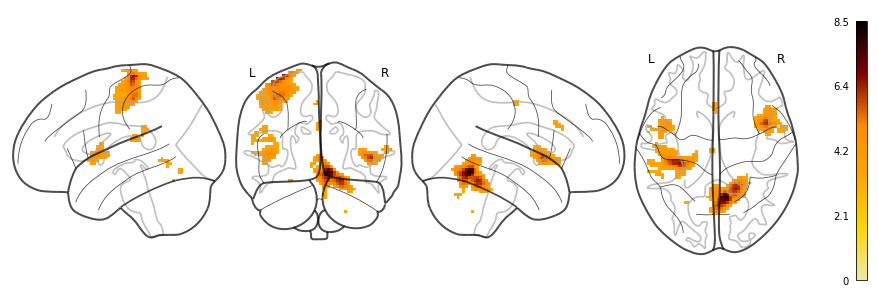


c) *playback (both hands)* > *playback (right hand)*


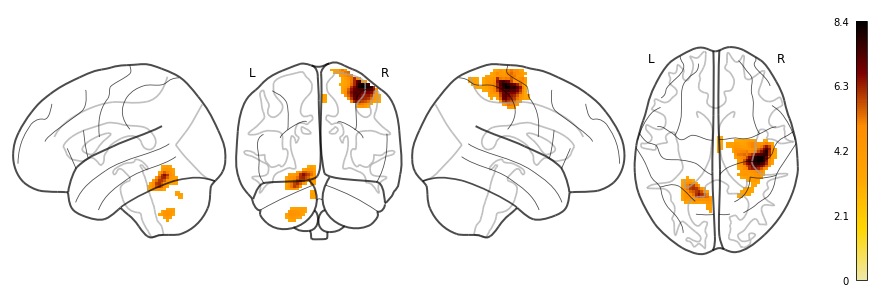


d) *altered auditory feedback* > *standard auditory feedback*


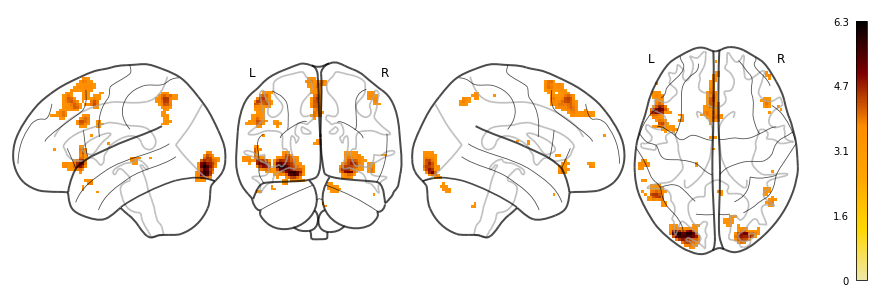


Fig. S8. Unthresholded statistical parametric maps for Task I (a, b, c) and Task II (d).

### S9: Figures corresponding to the methods and experimental design


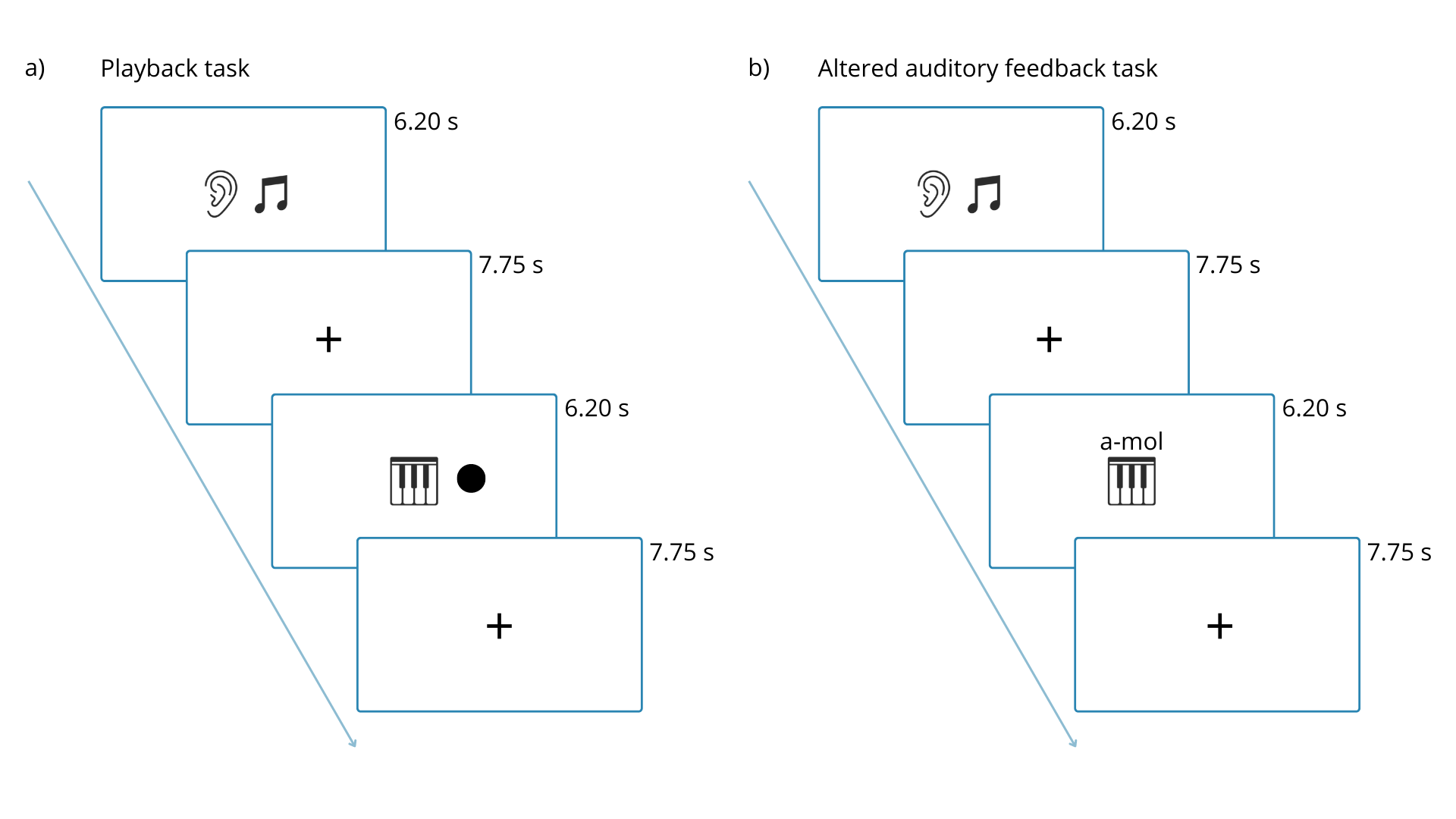


Fig. S9.1. A schematic representation of the experimental design. a) Task I - *listen* & *playback*. Participants are asked to play back exactly the excerpt they heard in the preceding *listen* trial, using one (a cue with a single dot as presented) or both hands (black dots on both sides). b) Task II - altered auditory feedback. Participants are asked to play back with their right hand a heptatonic scale they heard in the preceding *listen* trial and (the name of the scale is given in polish notation).


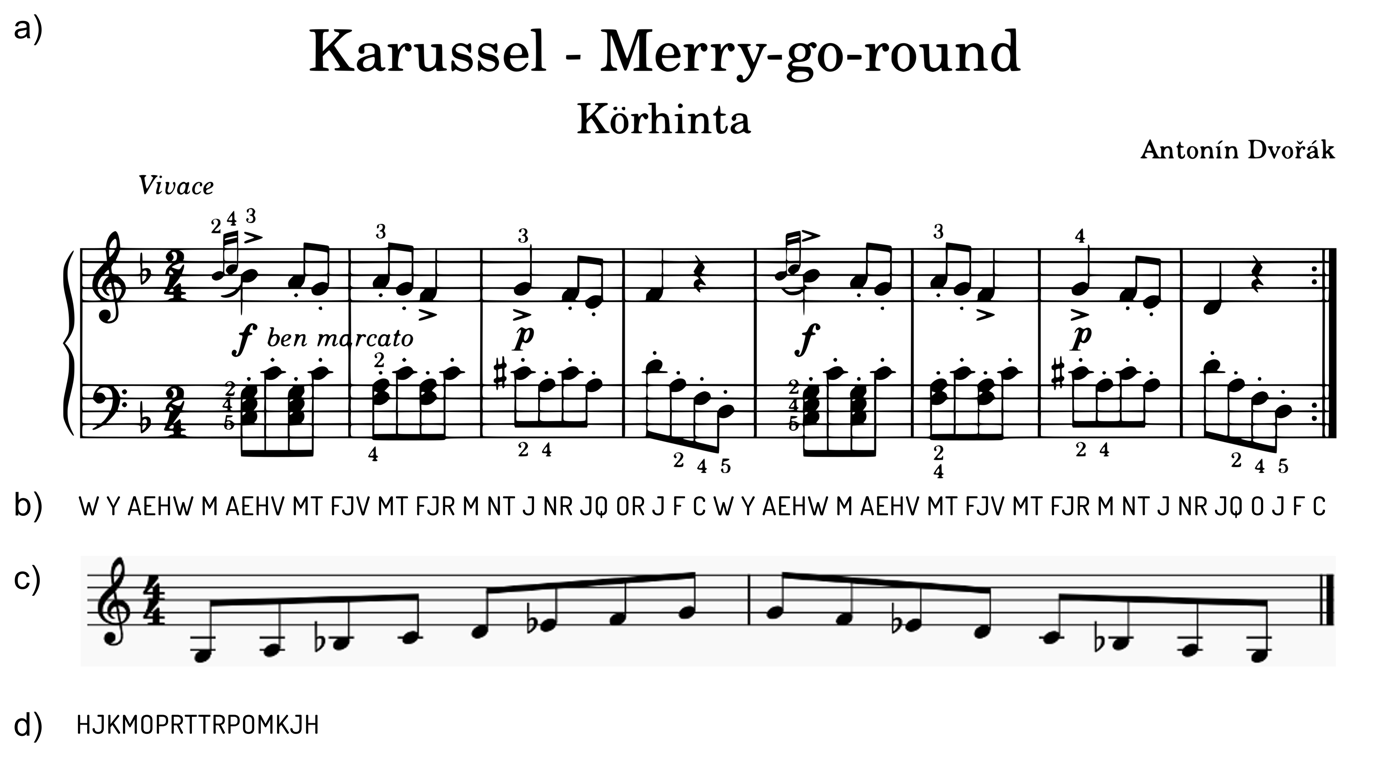


Fig. S9.2. Example stimuli and their encoding for Task I (a,b) and Task II (c,d): a) The original score for one of the stimuli (M06); b) The encoded score, with spaces separating the beats; c) score representation of the g-minor scale; d) the encoding of the g-minor scale. The original music by Antonín Dvořák is in the public domain; the transcription is original by the authors.

### S10: A comparison of movement (framewise displacement) between the listening and the playback experimental conditions

In order to test whether there was more movement during the playback trials as compared to the listening trials, we compared the framewise displacement (FD) between the two experimental conditions across all participants for both tasks using repeated-measures ANOVA. We found a small but significant increase in FD in the playback trials compared to the listening trials (p<0.001, F=16.391, η_p_^2^=0.462. M_listen_=0.257 SD=0.292, M_playback_=0.297 SD=0.312) in task I. However, we suggest that this difference is too small to be relevant, as it is in the sub-millimetre order of magnitude and does not exceed the voxel dimensions (2.5 mm). In task II, a similar absolute difference in FD is not significant (p=0.177, F=1.990, η_p_^2^=0.111. M_listen_=0.277 SD=0.335, M_playback_=0.300 SD=0.353). The comparison is visualised in fig. S10.


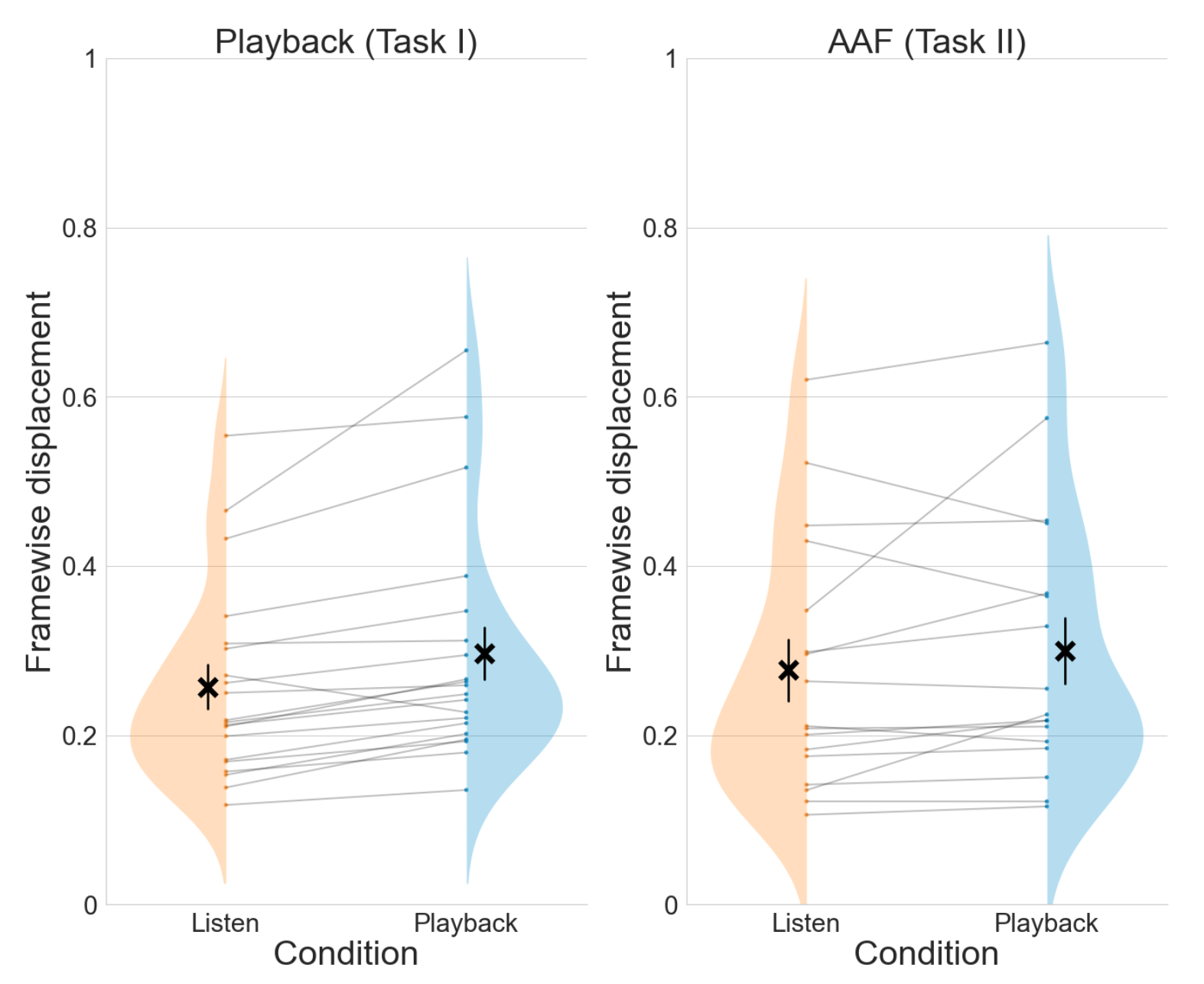


Fig.S10. Framewise displacement differences between the listen and the playback conditions for Task I and Task II. Lines represent the individual participants and dots represent the average FD per participant per condition; ✖ represent the mean per condition and bars represent the standard error of the mean (SEM).
